# Survival of the Replication Checkpoint Deficient Cells Requires MUS81-RAD52 Function

**DOI:** 10.1371/journal.pgen.1003910

**Published:** 2013-10-31

**Authors:** Ivana Murfuni, Giorgia Basile, Shyamal Subramanyam, Eva Malacaria, Margherita Bignami, Maria Spies, Annapaola Franchitto, Pietro Pichierri

**Affiliations:** 1 Section of Experimental and Computational Carcinogenesis, Department of Environment and Primary Prevention, Istituto Superiore di Sanità, Rome, Italy; 2 Section of Molecular Epidemiology, Department of Environment and Primary Prevention, Istituto Superiore di Sanità, Rome, Italy; 3 Department of Biochemistry, University of Illinois Urbana-Champaign, Urbana, Illinois, United States of America; 4 Department of Biochemistry, Carver College of Medicine, University of Iowa, Iowa City, Iowa, United States of America; University of Washington, United States of America

## Abstract

In checkpoint-deficient cells, DNA double-strand breaks (DSBs) are produced during replication by the structure-specific endonuclease MUS81. The mechanism underlying MUS81-dependent cleavage, and the effect on chromosome integrity and viability of checkpoint deficient cells is only partly understood, especially in human cells. Here, we show that MUS81-induced DSBs are specifically triggered by CHK1 inhibition in a manner that is unrelated to the loss of RAD51, and does not involve formation of a RAD51 substrate. Indeed, CHK1 deficiency results in the formation of a RAD52-dependent structure that is cleaved by MUS81. Moreover, in CHK1-deficient cells depletion of RAD52, but not of MUS81, rescues chromosome instability observed after replication fork stalling. However, when RAD52 is down-regulated, recovery from replication stress requires MUS81, and loss of both these proteins results in massive cell death that can be suppressed by RAD51 depletion. Our findings reveal a novel RAD52/MUS81-dependent mechanism that promotes cell viability and genome integrity in checkpoint-deficient cells, and disclose the involvement of MUS81 to multiple processes after replication stress.

## Author Summary

The replication checkpoint ensures a smooth duplication of the genome. It counteracts the replication stress, which can cause chromosome rearrangements as found in most tumours. Given the importance of dealing with perturbed replication, and since in tumours secondary mutations or epigenetic changes may hamper efficiency of the replication checkpoint, it is crucial to determine the mechanisms responding to replication perturbation upon checkpoint inactivation. Furthermore, it is highly relevant to understand how failure of these mechanisms correlates with chromosomal damage after replication perturbation. Here, we investigated pathways that, in checkpoint-deficient human cells, are involved in the handling of perturbed DNA replication forks, and we uncovered a previously unappreciated function of RAD52 and MUS81 in ensuring viability of cells, but at the expense of genome instability. We also demonstrated that checkpoint deficiency can trigger different mechanisms of recovery from replication arrest depending on the presence of RAD52 or MUS81, resulting in a poor survival and reduced genome instability or increased survival and chromosomal damage. Our work provides new clues about how human cells deal with replication stress, and how genome instability may arise in cancer cells.

## Introduction

Faithful completion of DNA replication and accurate transmission of the genetic information to daughter cells is of paramount importance. To ensure genome integrity, cells have evolved a sophisticated mechanism that supervises the replication process, the replication checkpoint [1]. Replication checkpoint is a system well conserved from lower to higher eukaryotes, and, in humans, is orchestrated by the ATR kinase [2]. ATR regulates directly or indirectly the function of several proteins involved in maintaining replisome stability, promoting restart of perturbed replication forks, and controlling cell cycle arrest [3]. The coordination of these activities is needed for completing replication, and avoiding accumulation of DNA damage or chromosomal rearrangements [4]. Consistently, replication checkpoint mutants fail to resume replication without accumulating DNA damage once the cause of the arrest is removed. These mutants also show chromosomal instability [1]. It has been suggested that inability of checkpoint mutants to resume replication at perturbed forks is directly related to their impaired capacity to stabilise them, eventually leading to accumulation of collapsed forks [1], [3]. Studies in yeast demonstrated that collapsed forks can be processed by exonucleases or converted into unusual replication intermediates, i.e. reversed forks, which can be substrates for endonucleases [5], [6], [7].

MUS81 is a structure-specific endonuclease that shows a remarkable preference for cleaving branched DNA substrates, such as nicked Holliday's Junctions (HJs), D-loops or three-way junctions [7], [8], [9]. MUS81 forms a heterodimeric complex with the non-catalytic EME1 subunit. Genetic studies in yeast have shown that this complex is involved in the resolution of HJs or in the processing of other replication intermediates generated at the perturbed forks [7], [9], [10]. In fission yeast, MUS81 is responsible for the formation of DNA double-strand breaks (DSBs), which are frequently observed in replication checkpoint mutants [11]. In addition, MUS81-dependent cleavage may take place downstream of RAD51 or RAD52 [12], [13]. In human cells, it has been shown that MUS81 is rapidly engaged at stalled replication forks to produce DSBs when fork collapse is triggered by loss of the Werner syndrome (WRN) RecQ helicase [14], [15], [16].

It remains unknown whether this function of MUS81 in human cells can be extended to other pathological conditions associated with replication checkpoint deficiency. Similarly, it is not known if cleavage by MUS81 in checkpoint-deficient cells occurs as a consequence of impaired, checkpoint-regulated RAD51 function [17]. Finally, the identity of the structure cleaved by MUS81 at stalled replication forks after checkpoint demise, as well as the mechanism underlying the fork collapse, remains undefined.

Here, we report that down-regulation of several replication-checkpoint factors inevitably leads to MUS81-dependent DSBs, which is essential to allow cellular recovery from replication stress. We also provide insights into the underlying mechanism by demonstrating that MUS81 cleavage is correlated to the loss of CHK1 activity, but is independent from the loss of RAD51 function. Moreover, we demonstrated that *in vitro* MUS81 acts on a D-loop formed by RAD52 but not RAD51, and that *in vivo*, after CHK1 inactivation, MUS81 functions downstream of RAD52. Our findings also suggest that loss of RAD52 promotes enhanced RAD51 chromatin association that is toxic in the absence of MUS81.

Altogether, these results provide an insight into how multiple mechanisms can cooperate at the distressed replication forks to allow cellular recovery, and viability in human cells with defective checkpoint function, a condition that may characterize a subset of human tumours and that may be exploited in targeted therapy.

## Results

### Down-regulation of replication checkpoint factors promotes MUS81-dependent accumulation of DSBs that are important for cell viability

**Figure 1:**
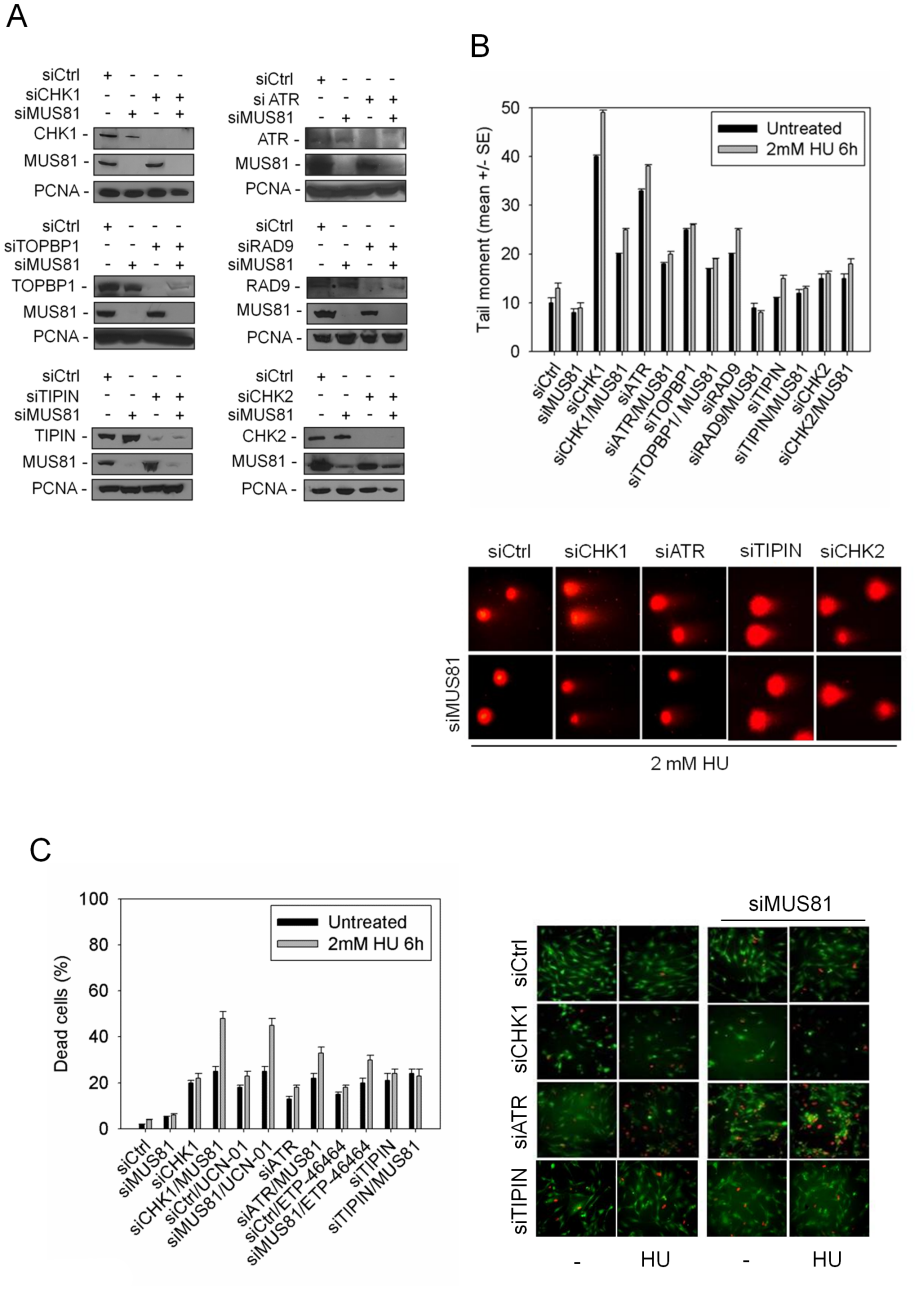
MUS81 promotes DSB formation and cell viability in response to replication checkpoint down-regulation. (**A**) Analysis of protein depletion by Western blotting in GM01604 cells after transfection with control siRNAs directed against GFP (siCtrl) or siCHK1, siATR, siTOPBP1, siRAD9, siTIPIN and siCHK2, alone or in combination with siMUS81. Immunoblotting was assessed 48 h after transfection using the appropriate antibodies. PCNA was used as loading control. (**B**) DSBs accumulation by neutral Comet assay in GM01604 cells transfected as in (A) and treated with 2 mM HU for 6 h before subjecting to Comet assay. Graph shows data presented as mean tail moment +/− SE from three independent experiments. Error bars represent standard errors. Where not depicted, standard errors were <15% of the mean. In the panel representative images from selected samples are shown. (**C**) Evaluation of cell death after replication stress in GM01604 cells transfected with control siRNAs (siCtrl) or siCHK1, siATR and siTIPIN alone or in combination with siMUS81. Forty-eight hours after RNAi, CHK1 inhibitor (UCN-01), ATR inhibitor (ETP-46464) or solvent (DMSO) was added to media 1 h prior HU treatment. After 6 h of HU, cells were recovered overnight before being analysed. Cell viability was evaluated by LIVE/DEAD assay as described in “Materials and Methods”. Data are presented as percentage of dead cells and are mean values from three independent experiments. Error bars represent standard error. Where not depicted, standard errors were <15% of the mean. The panel shows representative images: live cells are green stained, while dead cells are red.

In human cells, inactivation of replication checkpoint factors results in formation of DSBs [4]. To determine whether these DSBs are derived from MUS81-mediated endonucleolytic cleavage at stalled forks, we performed a neutral Comet assay after depletion of different checkpoint proteins. To this end, hTERT-immortalised human primary fibroblasts were transfected with siRNAs directed against selected checkpoint factors, by themselves or in combination with siRNAs against MUS81 (Figure 1A). In addition, we down-regulated expression of CHK2, a checkpoint kinase that is not activated after perturbed replication, and used siRNAs as internal controls. The efficiency of RNAi was evaluated by Western blotting 48 h post-transfection, and protein level of the targets was reduced by at least 80% in comparison to cells transfected with GFP siRNAs (siCtrl; Figure 1A). After transfection, cells were treated with 2 mM HU for 6 h, a condition sufficient to induce DSBs only under pathological condition [14], and subjected to neutral Comet assay. Depletion of each of the selected checkpoint factors resulted in DSBs formation when replication was not perturbed, and further enhanced after HU treatment (Figure 1B). The DSBs level was highest after CHK1 or ATR depletion, whereas RAD9 or TOPBP1 down-regulation resulted in a limited DSBs enhancement. No increase was observed in cells depleted of MUS81, TIPIN or CHK2. Interestingly, MUS81 depletion reduced DSBs levels, albeit to a different extent, in all the checkpoint-deficient cells, either untreated or treated with HU. The only exceptions were TIPIN or CHK2 knock-down cells (Figure 1B). Consistent results were also obtained analysing the formation of pan-nuclear staining of the phosphorylated H2AX. In this case, checkpoint impairment after ATR or CHK1 depletion gave rise to a strong accumulation of nuclei showing intense γH2AX staining, which was reduced by concomitant MUS81 knock-down (Figure S1A and B).

To investigate whether MUS81-dependent DSBs occurred during S-phase, cells released from G0-phase were transfected with siRNAs targeting CHK1, MUS81 or both, and then exposed to HU (Figure S2A). Suppression of DSBs by MUS81 depletion was detected in S-phase cells (Figure S2A and B). However, this was not simply due to an altered S-phase progression, since MUS81 knock-down did not induce premature G2 entry.

Upon oncogene-induced replication stress or in the absence of the WRN RecQ helicase, MUS81 function is required for cell viability [14], [18]. To examine whether MUS81-dependent DSBs were needed for cellular recovery in HU-treated replication checkpoint-deficient cells, we performed viability assays in cells in which ATR, CHK1 or TIPIN was down-regulated or chemically inhibited. As reported in Figure 1C, depletion of the selected checkpoint proteins enhanced cell death during both unperturbed and HU-perturbed proliferation. Interestingly, MUS81 down-regulation *per se* did not increase cell death, but hypersensitized ATR or CHK1 depleted cells to the replication arrest (Figure 1C). Moreover, even a short-term inhibition of ATR by ETP-46464 [19] or CHK1 by UCN-01 was sufficient to induce a MUS81 requirement for survival upon replication perturbation (Figure 1C). In contrast, MUS81 down-regulation did not synergize with loss of TIPIN (Figure 1C).

We next investigated whether enhancement of cell death by MUS81 depletion could reflect premature cell cycle progression. We analysed Bromo-deoxyuridine (BrdU) incorporation and arrest in G2-phase during recovery from HU in the cells in which MUS81 was down-regulated by RNAi, and the replication checkpoint was inhibited by UCN-01 or ETP-46464. To analyse replication recovery, cells were treated with HU, recovered for 60 or 120 min in drug-free medium, and exposed to BrdU for 30 min immediately before sampling. Both ATR and CHK1 inhibition caused a strong reduction in BrdU incorporation after HU, which was not reverted by prevention of MUS81-dependent DNA breakage (Figure S3A). Since inhibition of ATR or CHK1 results in accumulation of cells in G2-phase after over-night recovery from HU treatment [19], we analysed whether MUS81-depletion affected G2-phase arrest. For this purpose, cells enriched in S-phase by serum deprivation were transfected with Ctrl or MUS81 siRNAs, exposed to HU and recovered for 18 h. Western blotting analysis confirmed efficient depletion of MUS81 by RNAi after synchronisation (Figure S3B). As shown in Figure S3C, Ctrl RNAi cells showed a delayed S-phase upon HU treatment, and after an overnight recovery in the drug-free medium the cells accumulated in G2/M. Inhibition of CHK1 slowed further S-phase after HU, but MUS81 depletion did not rescue the phenotype (Figure S3C). Interestingly, MUS81 depletion delayed cell cycle progression after HU in mock-inhibited cells (Figure S3C).

Altogether, our observations indicate that down-regulation of replication checkpoint factors, but not of the DNA damage checkpoint kinase CHK2 or the replication fork protection factor TIPIN, results in MUS81-dependent DSBs. In addition, MUS81-dependent cleavage of stalled forks is required to maintain cell viability of replication checkpoint-deficient cells.

### MUS81-dependent DSBs at perturbed forks are due to CHK1 loss of function and not to an impaired RAD51

**Figure 2:**
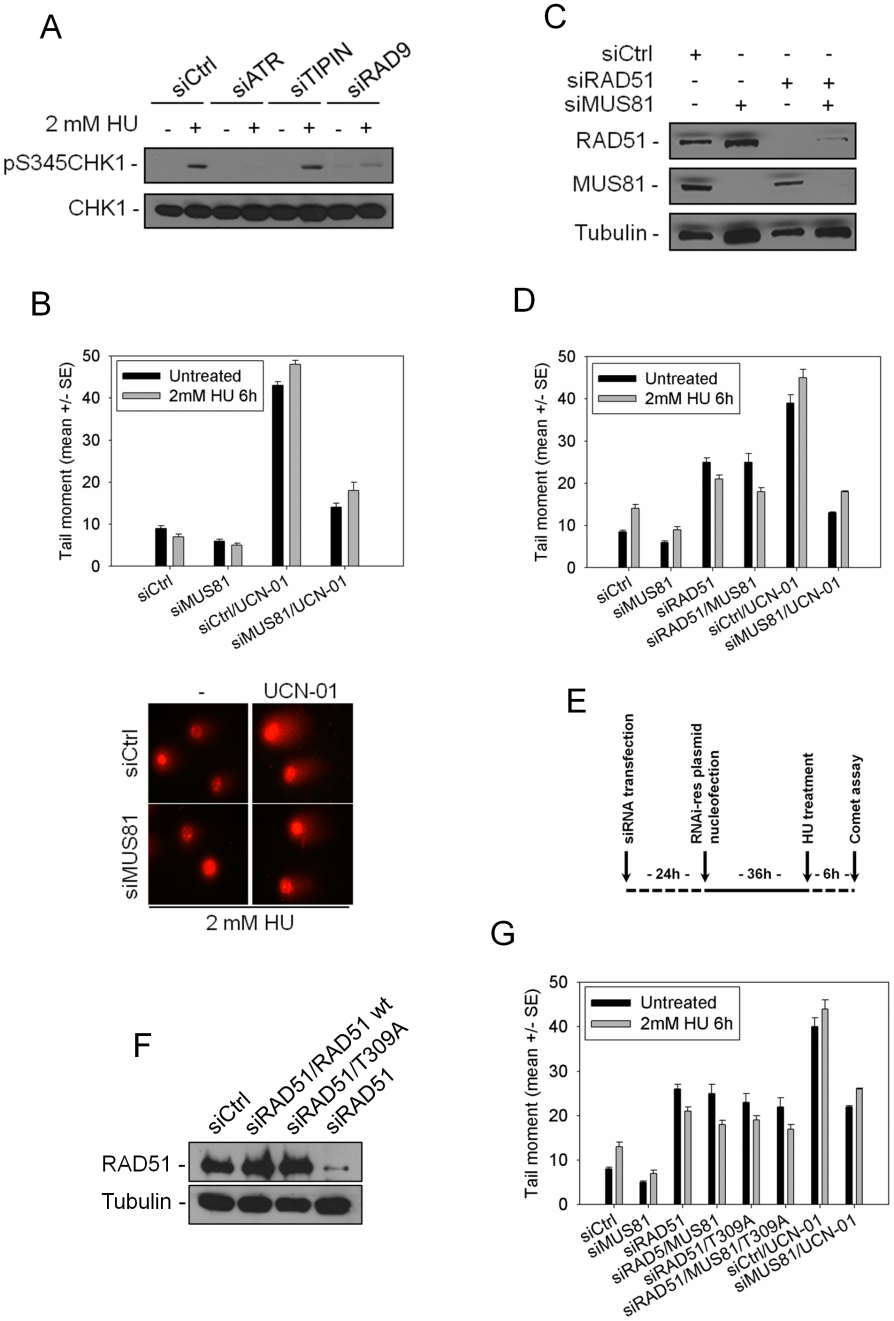
Loss of CHK1 function leads to MUS81-dependent DSBs production independently from RAD51 regulation. (**A**) Analysis of CHK1 phosphorylation in GM01604 cells transfected with control siRNAs (siCtrl) or siATR, siTIPIN and siRAD9 and treated with 2 mM HU for 6 h. Lysates were subjected to SDS-PAGE and immunoblotted for pS345CHK1 and CHK1. (**B**) Evaluation of MUS81-dependent DSBs formation by neutral Comet assay in cells in which CHK1 function was chemically inhibited. GM01604 cells were transfected with control siRNAs (siCtrl) or siMUS81. Forty-eight hours thereafter, cells were treated or not with CHK1 inhibitor (UCN-01) for 1 h and with 2 mM HU for 6 h and then subjected to Comet assay. Data are presented as mean tail moment and are means of three independent experiments. Error bars represent standard errors. Where not depicted, standard errors were <15% of the mean. In the panel, representative images are shown. (**C**) Western blotting in GM01604 cells transfected with control siRNAs (siCtrl) or siRAD51 and/or siMUS81. Depletion of proteins was verified 48 h after transfection using the relevant antibodies. Tubulin was used as loading control. (**D**) Analysis of DSBs formation in the absence of RAD51. Cells in which RAD51 and/or MUS81 was down-regulated were treated or not with UCN-01 for 1 h and with 2 mM HU for 6 h, then cells were subjected to neutral Comet assay. Cells treated with UCN-01 were used as positive control. Graph shows data presented as mean tail moment +/− SE from three independent experiments. Error bars represent standard errors. Where not depicted, standard errors were <15% of the mean. (**E**) Experimental scheme for genetic knock-down and rescue experiments. GM01604 cells were transfected with siRNA oligos targeting the UTR of RAD51 or GFP (siCtrl). RAD51-depleted cells were nucleofected to express RNAi-resistant wild-type or phosphorylation mutant form of RAD51 (RAD51-T309A). (**F**) Depletion of RAD51 and expression of the ectopic wild-type or RAD51-T309A were verified by immunoblotting 48 h thereafter using the anti-RAD51 antibody. Tubulin was used as loading control. (**G**) Analysis of DSBs formation in cells with impaired RAD51 function. GM01604 cells were transfected with control siRNAs (siCtrl) or siMUS81 and/or siRAD51. Forty-eight hours thereafter, cells were transfected with the RAD51-T309A plasmid (see Text S1). Then cells were treated or not with UCN-01 for 1 h and exposed to 2 mM HU for 6 h before being subjected to neutral Comet assay. Sample treated with UCN-01 was used as positive control. Data are presented as mean tail moment and are means of three independent experiments. Error bars represent standard errors. Where not depicted, standard errors were <15% of the mean.

Disruption of replication checkpoint function can lead to loss of CHK1 phosphorylation [4]. We observed the highest levels of DNA breakage after ATR or CHK1 silencing, while depletion of TIPIN, which may or may not affect CHK1 activation, did not induce MUS81-dependent DSBs. Thus, we investigated whether formation of DSBs by MUS81 was directly related to a defective CHK1 phosphorylation in checkpoint-deficient cells. To this aim, we down-regulated ATR, RAD9 or TIPIN, then we analysed CHK1 phosphorylation by Western blotting using phospho-specific antibodies. Down-regulation of ATR or RAD9, but not of TIPIN, impaired CHK1 phosphorylation at S345 (Figure 2A). It is worth noting that the residual level of CHK1 phosphorylation seems to be inversely correlated with the amount of MUS81-dependent DSBs. In fact, residual CHK1 phosphorylation in RAD9 RNAi cells corresponded to less DSBs than in ATR knock-down cells (see Figure 1B). CHK1 phosphorylation is a pre-requisite to kinase activation [20], and CHK1-mediated phosphorylation of downstream targets may contribute to preventing replication fork collapse *via* MUS81-dependent cleavage. Thus, cells in which MUS81 was down-regulated by RNAi were treated with UCN-01 to inhibit CHK1, alone or in combination with 2 mM HU, and then processed by neutral Comet assay. Inhibition of CHK1 by UCN-01 recapitulated the phenotype of CHK1 RNAi-treated cells, albeit with a reduced accumulation of DSBs (Figure 2B). However, MUS81 down-regulation decreased the number of DSBs formed as a consequence of UCN-01 treatment as efficiently as observed after CHK1 RNAi (Figure 2B and 1B). Chemical inhibition of CHK1 also allowed the analysis of time-dependent formation of DSBs at perturbed forks, as well as their genetic dependency on MUS81. UCN-01 triggered DSBs already after 4 h of HU treatment, and increased substantially at 6 h, when DSBs are detectable also in cells treated with UCN-01 or HU alone (Figure S4A). Similarly to what observed after 6 h of the combined UCN-01 and HU treatment, the DSBs detected at 4 h were MUS81-dependent (Figure S4B). Generation of DSBs by MUS81 could be secondary to accumulation of single-stranded DNA (ssDNA) regions or gaps at the leading or lagging strand, caused by the checkpoint inhibition. Using alkaline Comet assay, we analysed the formation of ssDNA regions or gaps after CHK1 inhibition at forks stalled by HU. As expected, in the HU-treated cells, ssDNA or gaps start to accumulate already at 1 h after CHK1 inhibition, and greatly increased at 4–6 h (Figure S4C), when DSBs (see Figure S4A) are also detected by alkaline Comet assay. Thus, formation of ssDNA regions or DNA gaps precedes MUS81-dependent cleavage at perturbed replication forks.

RAD51 is a CHK1 substrate, and its phosphorylation at T309 is required for recovery after HU stress [17]. Since it is unclear whether this phosphorylation is required for stabilizing stalled forks, we investigated the relation between MUS81-dependent cleavage of perturbed replication forks and the assembly of active RAD51 nucleoprotein filaments at ssDNA regions. In agreement with previous reports [17], we observed that depletion of ATR or chemical inhibition of CHK1 almost completely prevents RAD51 from nuclear foci assembly after HU treatment. In contrast, TIPIN down-regulation does not affect the ability of cells to relocalize RAD51 into foci (Figure S5).

Next, we analyzed whether loss of RAD51 phosphorylation and/or CHK1 activity equally promote MUS81 function. To this end, we analysed DSBs formation in cells depleted of RAD51 (Figure 2C). Consistently with other reports [21], [22], DSBs were detected after HU treatment. These DSBs, however, were not sensitive to MUS81 RNAi (Figure 2D). Consistently, analysis of DSBs in BRCA2 mutant cells revealed that MUS81 knock-down did not affect the level of DNA breakage induced by CHK1 inhibition and HU treatment (Figure S6). To further substantiate this observation, we transfected the RAD51 RNAi-depleted cells with an RNAi-resistant, wild-type RAD51 or the phosphorylation-defective RAD51-T309A mutant (Figure 2E). As shown in Figure 2F, the RNAi-resistant RAD51 proteins were expressed at levels comparable to the endogenous protein, and are not knock-downed by the siRNA oligo directed against the UTR of RAD51. The expression of the RAD51-T309A mutant also increased the amount of DSBs at levels similar to those associated with RAD51 loss (Figure 2G), and these breaks were abolished by the expression of a wild-type RAD51 (data not shown). Most importantly, DSBs observed in cells expressing the RAD51-T309A mutant were not reduced by depletion of MUS81 (Figure 2G). It is notable that loss of RAD51 function or phosphorylation results in DSBs levels substantially lower than in CHK1-deficient cells.

Altogether, these findings show that formation of MUS81-dependent DSBs in replication checkpoint-deficient cells is a consequence of the reduced CHK1 activity on targets distinct from RAD51. Furthermore, our results suggest that MUS81-dependent cleavage does not occur downstream of RAD51.

### DSBs in replication checkpoint-deficient cells are derived from MUS81-dependent cleavage of a substrate generated by RAD52

Since we showed that DSBs occurring in RAD51-depleted cells are independent from MUS81 function, we examined the possible involvement of RAD52 in generating a MUS81 substrate.

**Figure 3:**
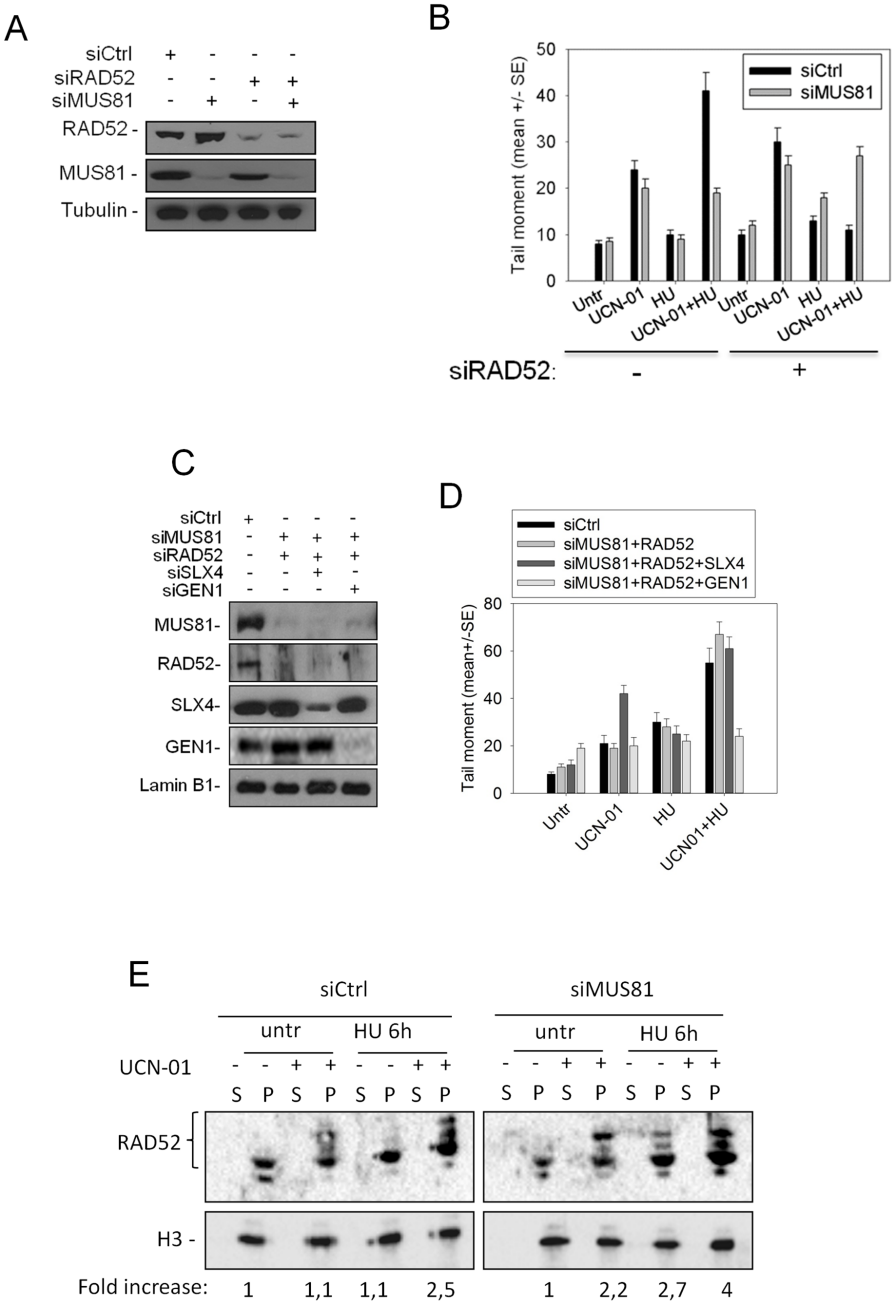
Role of RAD52 in MUS81-dependent DSBs formation. (**A**) Assessment of protein depletion by Western blotting in GM01604 cells after transfection with control siRNAs (siCtrl) or siRAD52 and/or siMUS81. Immunoblotting was performed using the relevant antibodies. Tubulin was used as loading control. (**B**) Analysis of DSBs accumulation in RAD52 depleted cells by neutral Comet assay. GM01604 cells were transfected as in (A) and treated with 400 nM UCN-01 and/or 2 mM HU for 6 h and then subjected to Comet assay. Graph shows data presented as mean tail moment +/− SE from three independent experiments. Error bars represent standard errors. (**C**) Assessment of protein depletion by Western blotting in GM01604 cells after transfection with control siRNAs (siCtrl) or the indicated combination of siRNAs. Immunoblotting was performed using the relevant antibodies. Lamin B1 was used as loading control. (**D**) Analysis of DSBs accumulation in RAD52/MUS81-depleted cells by neutral Comet assay. GM01604 cells were transfected as in (C) and treated with 400 nM UCN-01 and/or 2 mM HU for 6 h and then subjected to Comet assay. Graph shows data presented as mean tail moment +/− SE from three independent experiments. Error bars represent standard errors. (**E**) Levels of chromatin-bound RAD52 in GM01604 cells transfected with control siRNAs (siCtrl) or siMUS81 and treated with UCN-01 for 1 h and then with HU for 6 h. The amount of RAD52 in the chromatin fraction was presented as fold increase compared with the matched untreated control, normalized against the amount of histone H3.

Neutral Comet assays were performed in cells in which CHK1 was chemically inhibited and RAD52 depleted by RNAi alone or in combination with MUS81. Comparable reduction in protein levels was verified by Western blotting (Figure 3A). RAD52 down-regulation barely affected the level of DSBs after single treatments with HU or UCN-01 (Figure 3B). Upon combined treatment, however, RAD52 down-regulation efficiently suppressed DSBs, and this reduction was comparable to that observed following MUS81 depletion. In contrast, MUS81/RAD52 co-depletion resulted in the reappearance of DSBs, at levels similar to that of treatment with UCN-01 alone (Figure 3B). Even though either MUS81 or RAD52 down-regulation suppressed DSBs formation after CHK1 inhibition, MUS81 depletion strongly increased the mean tail moment observed by alkaline Comet assay, which are reversed by RAD52 but not RAD51 knock-down (Figure S7). Since the alkaline Comet assay detects both ssDNA or DNA gaps and DSBs, in the single depleted MUS81 or RAD52 cells, where DSBs are almost absent, only ssDNA regions are likely formed. In contrast, the reduction of the mean tail moment observed in the double MUS81/RAD52-depleted cells implies that only DSBs are formed.

To analyse whether DSBs generated in the absence of MUS81 and RAD51 might depend upon SLX4 or GEN1, two endonucleases that can substitute for MUS81 in processing DNA replication intermediates [23], [24], we analysed DSBs formation in the MUS81/RAD52 double-depleted cells in which each single endonuclease was down-regulated (Figure 3C). We found that, after UCN-01 and HU treatment, GEN1 depletion suppressed DSBs accumulation in the MUS81/RAD52-depleted cells (Figure 3D). Surprisingly, SLX4 down-regulation enhanced formation of DSBs in cells treated with UCN-01 alone (Figure 3D). Interestingly, whereas the DSBs produced by MUS81 in a wild-type background after UCN-01 and HU treatment are neither RAD51-independent nor repaired by RAD51, since RAD51 inhibition did not affect their appearance (Figure S8), DSBs formed by GEN1 depend on RAD51-mediated strand invasion (Figure S8).

We next analysed the recruitment of RAD52 to chromatin in cells treated with UCN-01 or HU, with or without prior MUS81 down-regulation. To this end, cellular fractionation experiments followed by SDS-PAGE and Western blotting were performed. In wild-type cells, chromatin localization of RAD52 did not change overtly after CHK1 inhibition or HU-induced replication arrest, however, the combined treatment increased three-fold the amount of chromatin-associated RAD52 (Figure 3E). This enhanced recruitment of RAD52 to chromatin was unaffected by MUS81 down-regulation (Figure 3E).

Together, analyses of DSBs and RAD52 chromatin association indicate that MUS81 and RAD52 cooperate in the replication checkpoint-deficient cells, and that RAD52 may work upstream of MUS81.

**Figure 4:**
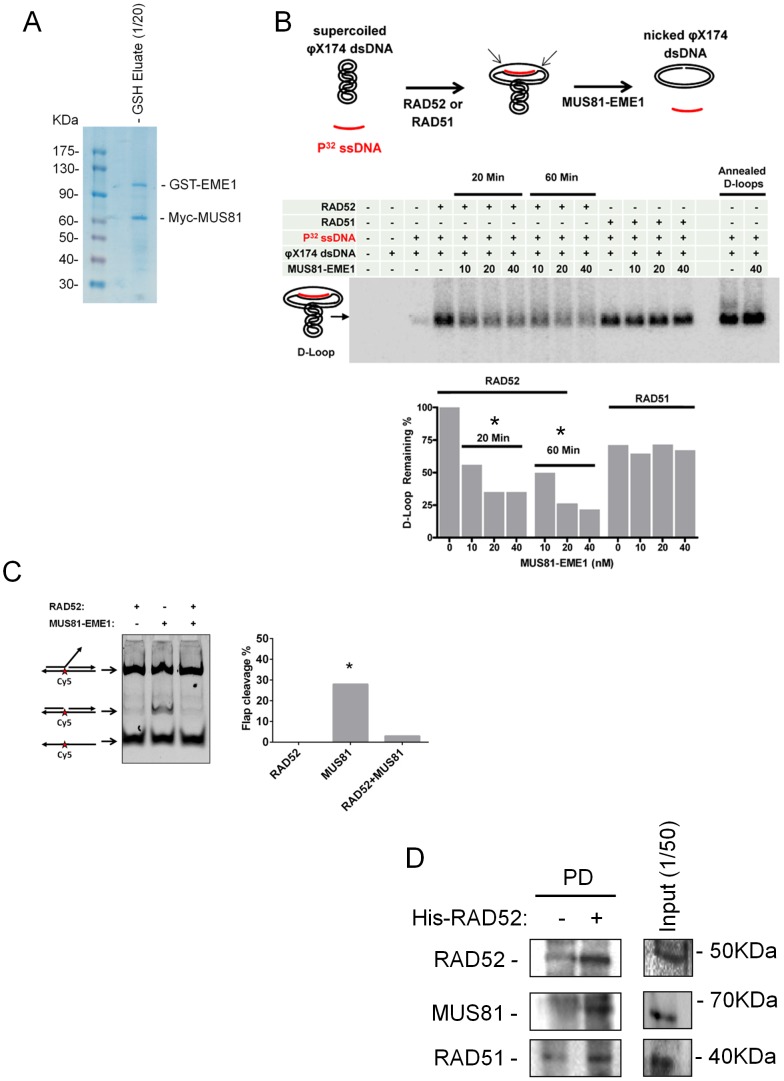
(A) Immunopurification of the human MUS81/EME1 complex from 293T cells. One-twentieth of human MUS81/EME1 complex immunopurified using anti-Myc-agarose/GSH agarose (see Materials and Methods) was resolved onto an SDS-PAGE gel and revealed by Coomassie blue-stain (CBB). (**B**) In vitro MUS81/EME1 cleavage of model D-loop substrates. The D-loops were produced by the RAD52-mediated annealing, by the RAD51-mediated strand invasion, or by the heat-mediated annealing as described in “Materials and Methods” and schematically depicted over the gel. MUS81/EME1-mediated cleavage results in the loss of superhelicity and, upon deproteination of the products, in the displacement of the radioactively-labeled oligonucleotide from the plasmid. Thus, the D-loop loss is an indicator of MUS81/EME1-dependent D-loop cleavage. The D-loops were separated from the unincorporated and displaced oligonucleotides on the agarose gel. The table above the gel summarizes the constituencies and conditions of each reaction. The band corresponding to the D-loop migration is marked on the side of the gel. The graph under the gel shows the gel quantification. (**C**) In vitro MUS81/EME1 cleavage of a 3′-flap substrate. The substrate was assembled as described in “Materials and Methods” and schematically depicted side the gel. MUS81/EME1-mediated cleavage results in formation of a nicked product, which was separated from the intact substrate and the not-assembled, single-stranded, substrate on the agarose gel. The graph shows the gel quantification. (**D**) RAD52 pulled-down MUS81 from nuclear extracts. Five µg of purified 6xHis-tagged RAD52 was incubated with 1 mg of benzonase-treated nuclear extract. After incubation with anti-His antibody-coupled magnetic beads, RAD52 protein complexes were released in 1x Laemmli sample buffer, subjected to SDS-PAGE and Western blotting using the indicated antibodies. Data are presented as a mean of replicate experiments, SEs were <10% of the mean. * = p<0.05 Student's t-test.

One of the putative MUS81 substrates that may be formed at the HU-stalled or collapsed forks is a D-loop [7]. To verify this possibility, we evaluated the ability of a purified MUS81/EME1 complex to cleave a model D-loop assembled *in vitro* by either RAD52 or RAD51. The human MUS81/EME1 complex was immunopurified from 293T cells transiently transfected with plasmids expressing Myc-tagged MUS81 and GST-tagged EME1 (Figure 4A). Purified RAD52 or RAD51 were pre-incubated with P^32^-labelled oligonucleotides complementary to a region of the φX174 plasmid. The resulting nucleoprotein complexes mediated formation of the D-loops comprised by φX174 RFI supercoiled dsDNA and the P^32^-labelled oligonucleotides. The resulting D-loops were incubated with increasing amounts of the MUS81/EME1 complex. Cleaving or nicking the D-loop should result in the loss of superhelicity, displacement of the oligonucleotide, and disappearance of the D-loop. As shown in Figure 4B, MUS81/EME1 cleaved the D-loop produced by RAD52 in a concentration-dependent manner, as demonstrated by the reduction in the amount of the substrate. In contrast, the D-loop produced by RAD51-mediated strand invasion appeared resistant to endonucleolytic cleavage (Figure 4B). Notably, cleavage of this type of a D-loop required the presence of both MUS81/EME1 nuclease and RAD52 since MUS81/EME1 was extremely less efficient in cleaving the protein-free D-loops produced in the control experiment by heat-mediated annealing. To investigate if RAD52 stimulation of MUS81 activity was specific for the D-loop, we prepared a Cy5-labelled 3′-flap substrate, which represents one of the acknowledged and preferred MUS81 substrates. As shown in Figure 4C, MUS81 cleaved the 3′-flap substrates, giving rise in the generation of the nicked product. As expected, incubation of RAD52 alone did not result in any cleavage but, surprisingly, it prevented almost completely MUS81 from cutting the ssDNA flap.

Next, we investigated whether RAD52 and MUS81 do physically interact, performing a pull-down assay using purified recombinant RAD52 as bait and HeLa nuclear extracts, which were treated with a nuclease to get rid of any DNA-bridged interactions. As Figure 4D shows, recombinant RAD52 pulled-down MUS81 from the nuclear extracts. As expected, RAD51, which is known to associate with RAD52, was also found in the RAD52 pull-down.

Collectively, our results suggest that, after inhibition of CHK1 activity, loading of RAD52 in chromatin increased leading to the formation of an intermediate, likely a D-loop, which is cleaved by MUS81. In the absence of both RAD52 and MUS81, however, DSBs occur as a result of GEN1-dependent cleavage downstream of RAD51. These results also suggest that RAD51 and RAD52 may compete at the fork, and that RAD52 may be recruited at collapsed forks independently of MUS81.

### Reduced replication restart observed after CHK1 inhibition can be reverted by RAD52 down-regulation

**Figure 5:**
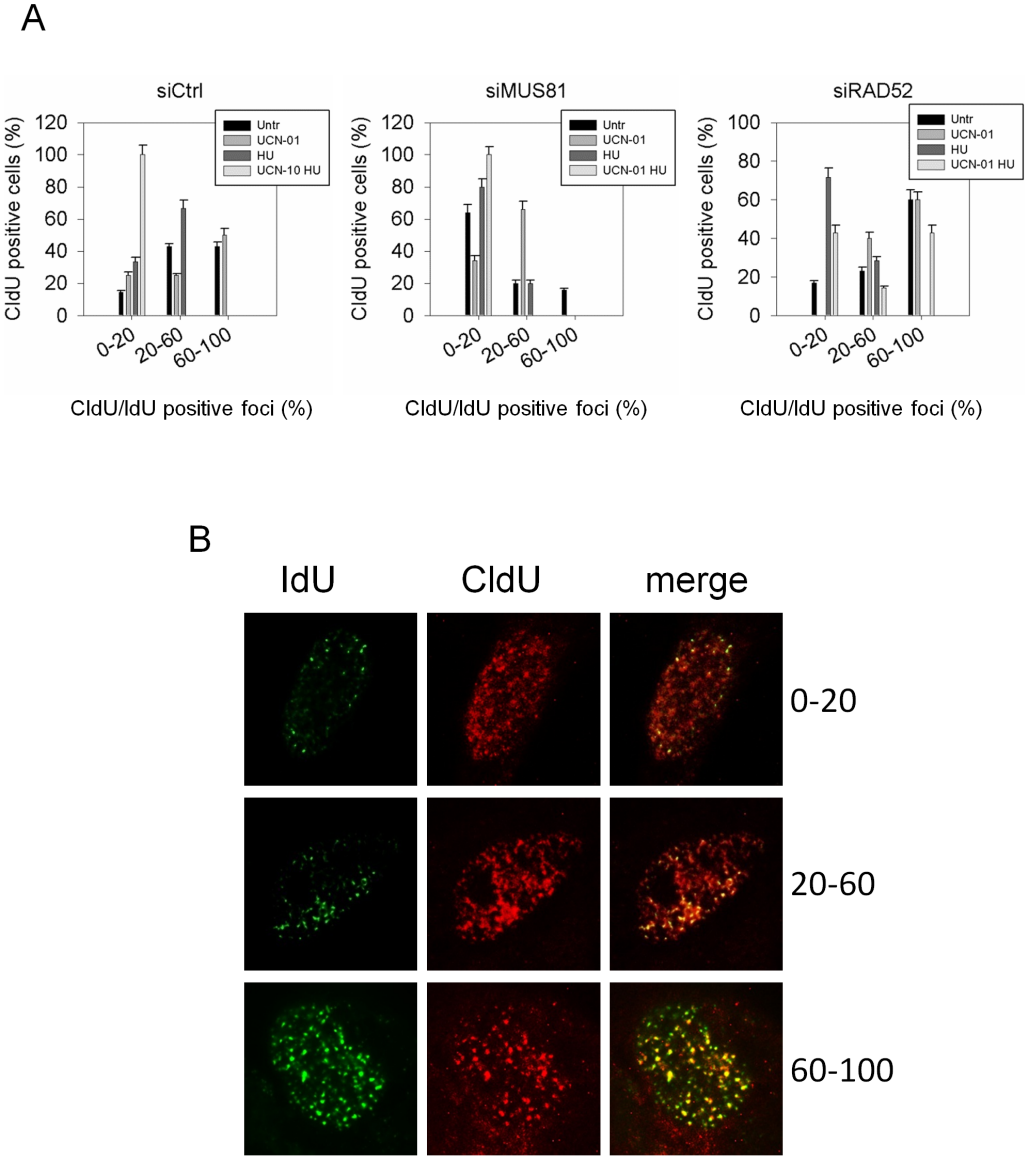
MUS81 and RAD52 differently affect restart of stalled forks upon CHK1 inhibition. (**A**) GM01604 cells were transfected with siRNAs directed against GFP (siCtrl), MUS81 (siMUS81) or RAD52 (siRAD52). Replication sites were first labeled with CldU (red signal), left untreated or treated as indicated, followed by recovery for 45 min in IdU (green signal). After immunostaining with antibodies specific for CldU and IdU, the overlapping foci were quantified in each isolated red-positive cell and results expressed as the percentage of CldU/IdU colocalising foci (0–20% of total CldU foci; 20–60% of total CldU foci; 60–100% of total CldU foci). Data are presented as percentage of dead cells and are mean of three independent experiments. Error bars represent standard error. The images shown in the panel (**B**) are representative of labeling and of different classes of colocalising nuclei.

MUS81 has been involved in replication restart after prolonged replication inhibition [25]. Having shown that, in the absence of CHK1 function, RAD52 and MUS81 cooperate in the formation of DSBs at stalled forks, and that their function is required to ensure viability of replication-stressed checkpoint-deficient cells, we studied their relationship with restart of such collapsed forks. Using a double CldU/IdU labelling approach on interphase nuclei, we examined the ability of MUS81 or RAD52-depleted cells to restart replication forks [18]. MUS81 or RAD52 down-regulation did not reduce the number of cells that incorporate the first label (CldU) as compared to the wild-type cells (siCtrl and data not shown), but differently affected incorporation of the second label (IdU) at active replication factories (Figure 5A and B). As expected, CHK1 inhibition severely decreased the ability of HU-treated cells to restart DNA synthesis at stalled forks, as evidenced by the absence of nuclei with more than 60% of CldU/IdU colocalizing foci (Figure 5A and B). A reduction of the ability to incorporate the second label was also observed in MUS81-depleted cells in both unperturbed and HU-exposure conditions (Figure 5A and B). In cells treated with UCN-01 and HU, MUS81 down-regulation did not modify the extent of restart at active replication foci (Figure 5A and B). Interestingly, in RAD52-depleted cells, the increased number of CldU-positive nuclei in which IdU is incorporated at active nuclear foci, demonstrates that HU-stalled forks were recovered in the presence of UCN-01 (Figure 5A and B). Moreover, in UCN-01 and HU-treated cells, RAD52 down-regulation apparently enhanced also the number of nuclei showing only IdU-positive replication foci, which were barely detectable in MUS81-depleted cells (data not shown).

Altogether, these results indicate that MUS81 does not contribute to the reduction of fork restart caused by CHK1 inhibition, and that this impairment is mostly dependent on the activation of RAD52.

### Loss of RAD52 and MUS81 leads to the accumulation of toxic RAD51-dependent intermediates resulting in cell death upon CHK1 inhibition

**Figure 6:**
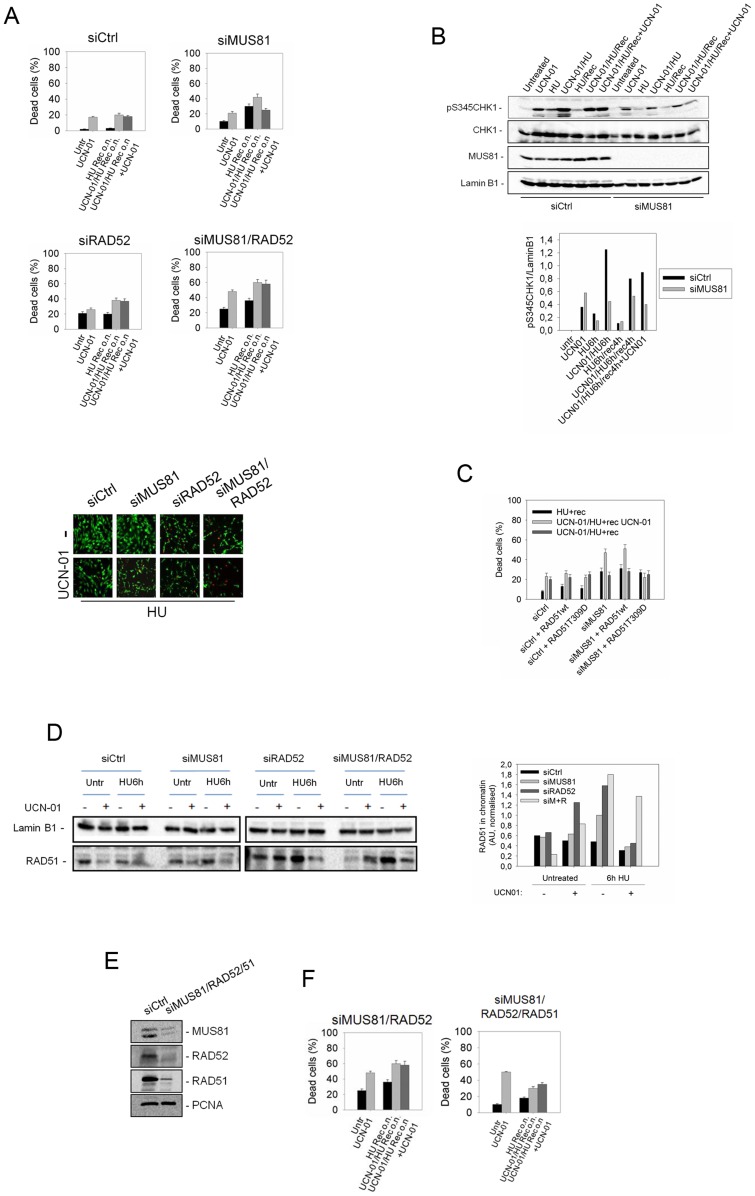
MUS81 and RAD52 promote survival also independently from each other. (A) Effect of the down-regulation of RAD52 and/or MUS81 on cell viability. GM01604 cells were transfected with the indicate siRNAs and 48 h later treated with 400 nM UCN-01 and/or 2 mM HU for 6 h. Cell viability was evaluated by LIVE/DEAD assay as described in “Materials and Methods” after 18 h of recovery in HU-free medium, with or without continuous exposure to UCN-01. Data are presented as percentage of dead cells and are mean of three independent experiments. Error bars represent standard error. Where not depicted, standard errors were <15% of the mean. In the panel representative images from samples treated with HU are reported: live cells are green stained while dead cells are red. (**B**) Analysis of replication checkpoint activation. Cells were treated with 400 nM UCN-01 and/or 2 mM HU for 6 h. Then cells were recovered for 4 h and immunoblotted for pS345CHK1 and CHK1. MUS81 was used to verified protein depletion and Lamin B1 as loading control. The graph shows the gel quantification. (**C**) Effect of the over-expression of RAD51-T309D on cell viability of cells experiencing replication stress in the absence of MUS81. GM01604 cells were transfected with the indicate siRNAs and 24 h later nucleofected with plasmids expressing either RAD51wt or RAD51-T309D. Twenty-four hours thereafter, cells were treated with 400 nM UCN-01 and/or 2 mM HU for 6 h. Cell viability was evaluated by LIVE/DEAD assay as described in “Materials and Methods” after 18 h of recovery in HU-free medium, with or without continuous exposure to UCN-01. Data are presented as percentage of dead cells and are mean of three independent experiments. Error bars represent standard error. Where not depicted, standard errors were <15% of the mean. (**D**) Levels of chromatin-bound RAD51 in GM01604 cells transfected with the indicated siRNAs. Forty-eight hours after transfection, cells were treated with UCN-01 for 1 h and then with HU for 6 h. The graph shows the amount of RAD51 in the chromatin fraction determined after densitometry of the representative gels and presented as arbitrary units normalized against the amount of Lamin B1. (**E**) Western blotting showing depletion of protein levels after transfection with the indicated siRNAs. PCNA was used as loading control. (**F**) Effect of the down-regulation of RAD51 in RAD52/MUS81-depleted cells on cell viability. GM01604 cells were transfected with control siRNAs (siCtrl) or siRAD52, siRAD51 and siMUS81. Forty-eight hours after interference, cells were treated with 400 nM UCN-01 and/or 2 mM HU for 6 h. Cell viability was evaluated by LIVE/DEAD assay 18 h after recovery in HU-free medium, as described in “Materials and Methods”. Data are presented as percentage of dead cells and are mean of three independent experiments. Error bars represent standard error. Where not depicted, standard errors were <15% of the mean.

To address whether RAD52 might also play a MUS81-independent role in cells with a compromised CHK1 function, we evaluated cell death after recovery from replication stress in cells depleted of MUS81, RAD52 or both, with or without persistent CHK1 inhibition. We found that, in wild-type cells, combined exposure to HU and UCN-01 resulted in a 20% cell death. When cells were allowed to recover in the absence of the CHK1 inhibitor, only a minimal reduction in toxicity was observed (Figure 6A). After CHK1 inhibition, cell death of MUS81-depleted cells increased by two-fold, but decreased significantly when UCN-01 was left during recovery (Figure 6A). Among other enzymes involved in the resolution of intermediates thought to accumulate at collapsed forks, i.e. SLX4, GEN1 or BLM, only GEN1 depletion increased cell death in CHK1-deficient cells after HU treatment (Figure S9A). RAD52 down-regulation also resulted in enhanced cell death during recovery from UCN-01-induced replication stress. This phenotype, however, was unaffected by persistent CHK1 inhibition during recovery (Figure 6A). Interestingly, the simultaneous inactivation of RAD52/MUS81 was associated with extreme toxicity. Indeed, cells depleted of RAD52 and MUS81 showed about 60% cell death, independently of CHK1 activity during recovery (Figure 6A). Increased cell death of MUS81-depleted cells was also observed following down-regulation of GEN1 or SLX4, and at a lesser extent after BLM RNAi (Figure S9B), suggesting their involvement in processing intermediates formed at stalled forks in the absence of MUS81.

These results show that MUS81-depleted cells are protected from replication stress induced by CHK1 inhibition only when CHK1 function is restored during recovery. To reinforce this conclusion, we verified whether CHK1 activation was enhanced in MUS81 knock-down cells after recovery from replication stress induced by checkpoint impairment. Following exposure to HU and UCN-01, CHK1 phosphorylation at Ser345, a diagnostic readout of its activation, was clearly enhanced (Figure 6B). The levels of Ser345-phosphorylated CHK1 were, however, greatly reduced by MUS81 depletion, suggesting that breakage at collapsed forks is responsible for further checkpoint signalling (Figure 6B). Recovery from replication stress also reduced CHK1 phosphorylation in wild-type cells, even though the residual level of phosphorylated CHK1 is unaffected by UCN-01 removal (Figure 6B). In contrast, phosphorylation level of CHK1, even though lower, did not decrease during recovery in MUS81 RNAi cells as compared with Ctrl RNAi cells (Figure 6B). Since, in the absence of MUS81, CHK1 phosphorylation was maintained during recovery from replication stress, we tested the possibility that this was due to prolonged checkpoint activation. However, we found that was not the case (Figure S2C).

Thus, we hypothesized that CHK1 activation was required to sustain RAD51 function. We reasoned that overexpression of the phosphomimetic RAD51-T309D mutant in cells depleted of MUS81 would ameliorate viability independently from the presence of UCN-01 during recovery. Consistently, by overexpressing the RAD51-T309D mutant, cell survival was strikingly increased during recovery from replication arrest, irrespectively of CHK1 inhibition (Figure 6C). In contrast, overexpression of wild-type RAD51 protein did not modify the toxic response induced by MUS81 depletion, and its dependence on CHK1 inhibition (Figure 6C). Finally, overexpression of the RAD51-T309D mutant did not rescue the elevated cell death observed in the RAD52 or in the RAD52/MUS81-depleted cells, but instead exacerbated the phenotype of the double knock-down cells (data not shown).

Since, in human cells, RAD52 may be required for RAD51 chromatin loading under perturbed replication [26], we analysed whether RAD52 down-regulation affected RAD51 recruitment after CHK1 inhibition and HU treatment. Analysis of chromatin fractions from cells treated with HU or UCN-01 showed that the amount of RAD51 in chromatin did not increase, and only a small reduction was observed after a concomitant treatment (Figure 6D). We observed that the amount of RAD51 chromatin-bound is higher in HU-treated MUS81 knock-down cells than in Ctrl RNAi cells, but decreases after a combined exposure of HU and UCN-01 (Figure 6D). Interestingly, the loading of RAD51 was unaffected by RAD52 depletion in untreated cells, and was greatly increased after UCN-01 or HU treatment (Figure 6D). Co-depletion of RAD52 and MUS81 determined also a strong increase in the level of chromatin-associated RAD51 after replication arrest by HU (Figure 6D), which was maintained in the presence of the CHK1 inhibitor (Figure 6D).

Elevated chromatin accumulation of RAD51 and the hypersensitivity of the RAD52/MUS81 knock-down cells to replication stress induced by the a combined HU and UCN-01 exposure, prompted us to verify whether exacerbated cell death was related to the inability to properly process RAD51-dependent intermediates. To this aim, we analysed whether RAD51 depletion could improve viability of the double RAD52/MUS81 knock-down cells after replication stress. Interestingly, concomitant depletion of RAD51, RAD52 and MUS81 (Figure 6E) reduced significantly cell death in cells treated with UCN-01, alone or in combination with HU, irrespectively of the presence of CHK1 activity during recovery (Figure 6F). The severe phenotype of the RAD52/MUS81 double-depleted cells was also ameliorated by GEN1 down-regulation, but not by SLX4 depletion (Figure S10A). In contrast, GEN1 down-regulation did not rescue, but rather reduced, viability of the RAD52 single-depleted cells (Figure S10B).

Our results indicate that a CHK1-regulated RAD51 function can prevent the MUS81-dependent cell death derived from replication stress, induced by CHK1 inhibition. Moreover, our findings also suggest that loss of RAD52 engages a RAD51-dependent recovery in which MUS81 may play an important and additional function, together with BLM, to clear potentially toxic intermediates.

### RAD52/MUS81-dependent processing of collapsed forks enhances chromosomal damage in CHK1-deficient cells

**Figure 7:**
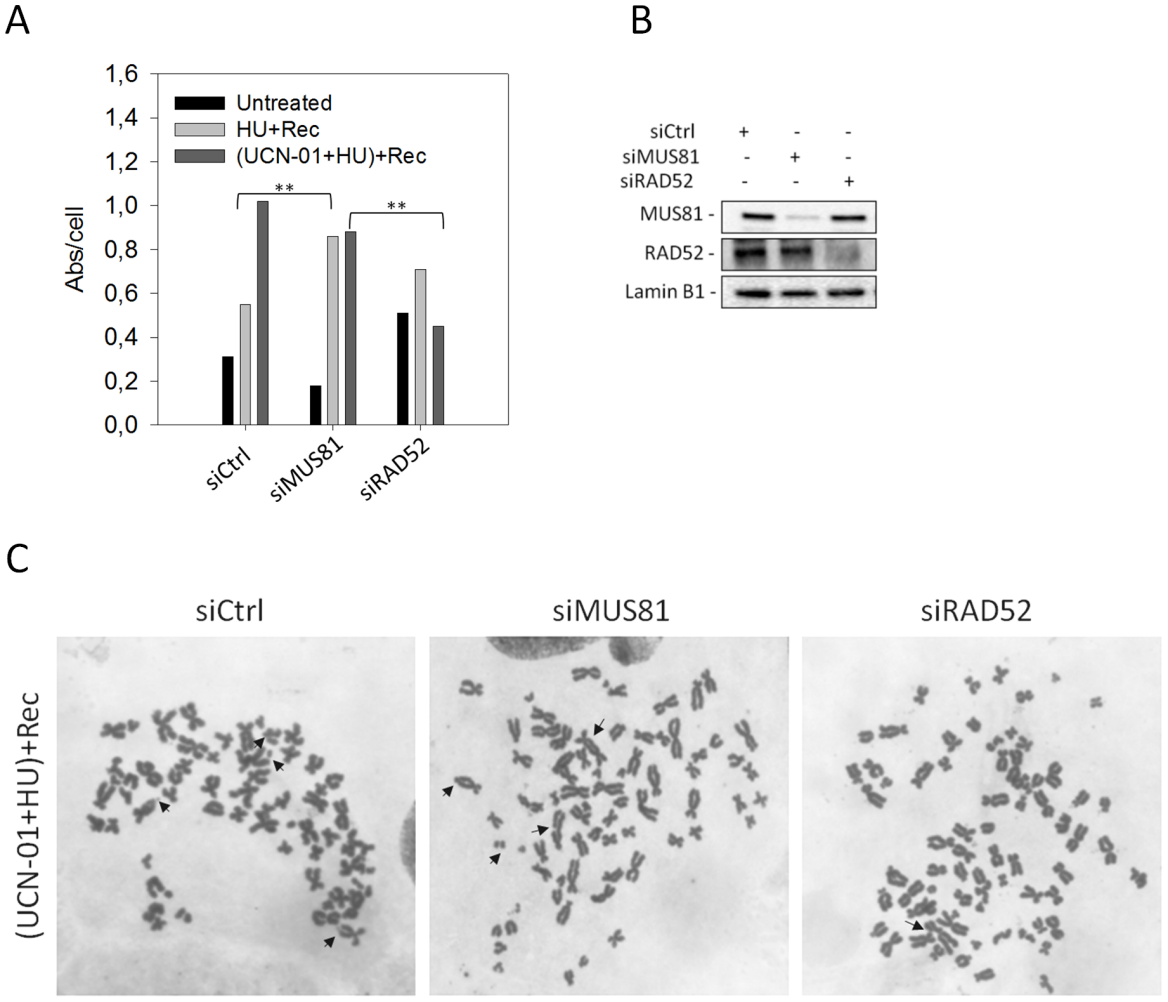
Effect of MUS81 or RAD52 depletion on chromosomal damage in response to replication checkpoint down-regulation. (**A**) Western blotting showing MUS81 and RAD52 depletion verified 48 h after interference using the relevant antibodies. Lamin B1 was used as loading control. (**B**) Aberrations per cell in WI-38 SV40-transformed fibroblasts transfected with control siRNAs (siCtrl), siMUS81 or siRAD52. Cells were treated as described in “Materials and Methods”. Asterisks indicate that the result is statistically significant compared to the indicated experimental point; (** = P<0.05, Student's *t* test). (**C**) Representative Giemsa-stained metaphases from cells transfected with the indicated siRNAs and recovered in drug-free medium after replication checkpoint inhibition. Arrows indicate chromosomal aberrations.

We have previously shown that loss of MUS81 increases chromosomal damage in WRN-deficient cells, whereas it decreases chromosome abnormalities upon oncogene-induced replication stress [14], [18]. In both cases, however, MUS81 down-regulation increases cell death as we observed in replication checkpoint-deficient cells. Thus, we investigated whether MUS81 down-regulation enhanced chromosomal instability in CHK1-inhibited cells. To this end, we induced replication stress by concomitant CHK1 inhibition and HU treatment, and analysed chromosomal damage in metaphase cells after recovery in the absence of UCN-01 to limit cell death. Given that double knock-down cells were extremely sick, we limited the analysis of chromosome damage in Ctrl, MUS81 or RAD52 RNAi-treated cells (Figure 7A–C).

A combined UCN-01 and HU treatment resulted in a significant increase of chromosome aberrations, mainly chromosome breaks, compared to HU-treated cells (Figure 7A, C). Depletion of MUS81 enhanced chromosome damage in cells treated with HU alone, while barely affected genome instability caused by the combined treatments (Figure 7B, C). RAD52 down-regulation resulted in increased levels of chromosomal damage in both unperturbed and HU-exposure conditions. In contrast, a significant reduction in chromosome aberrations was observed in cells treated with UCN-01 and HU (Figure 7A, C).

These results indicate that most of the chromosomal damage, resulting from checkpoint failure, is the result of the engagement of RAD52 and MUS81 at collapsed forks, which would grant viability, but at the expense of genome stability.

## Discussion

### Inactivation of CHK1 induces MUS81-dependent DSBs at perturbed forks to support viability under replication stress

One of the essential functions of the replication checkpoint is to maintain integrity of replication forks when they undergo pausing or stalling. Consistently, studies from model organisms and human cells with impaired replication checkpoint activity have shown elevated levels of collapsed forks and DSBs accumulation after replication perturbation [27], [28], [29], [30], [31], [32]. Our data indicate that MUS81 is responsible for the creation of DSBs after depletion of some crucial components of the replication checkpoint, extending recent findings indicating that MUS81 acts in the cells with mutant WRN protein, or following replication stress induced by oncogene activation or camptothecin treatment [14], [18], [25], [33]. In fission yeast, an analogous MUS81-dependent formation of DSBs has been reported [11], suggesting that the function of MUS81 at the collapsed forked is conserved across species. Despite the number of functions that the replication checkpoint fulfils upon replication stalling [1], here we demonstrate that loss of CHK1 activity is sufficient to cause MUS81-mediated DSBs. This agrees with earlier observations showing that depletion of ATR, RAD9 or TOPBP1 results in a reduced CHK1 phosphorylation [20], [34], [35], [36], and DSBs suppression following MUS81 down-regulation in unperturbed CHK1-inhibited cells [37]. Although it has been shown that a proper CHK1 activation requires the presence of additional factors, such as TIPIN [38], [39], we observed no accumulation of MUS81-dependent DSBs in TIPIN knock-down cells after HU treatment. Given that, in our cell model system, TIPIN down-regulation does not reduce CHK1 phosphorylation, and since TIPIN/TIM-deficient cells retain the ability to sustain CHK1 activation [40], it is possible that even a reduced amount of active CHK1 is sufficient to protect from MUS81-dependent DSBs.

It has been recently reported that DSBs induced by MUS81 are detrimental to cell survival in CHK1 inhibited cells [37]. In contrast, we show that MUS81-dependent DSBs are essential to limit cell death upon replication stress induced by HU treatment and CHK1 inhibition. Moreover, another structure-specific endonuclease, GEN1, is also required to prevent excessive cell death in CHK1-inhibited cells experiencing replication arrest. However, almost all the DSBs formed are MUS81-dependent under our experimental conditions. Thus, the observed pro-survival role of GEN1 might be related to resolution of late homologous recombination (HR) intermediates, rather than to cleavage at collapsed forks, as recently proposed [41]. These results are not necessarily in conflict with the observations of Forment and colleagues, since only untreated cells have been analysed. Moreover, our results are in agreement with previous reports indicating that DSBs produced by MUS81 are required to allow replication recovery, and viability, under different conditions resulting in fork collapse [11], [14], [25], [33].

### MUS81-dependent DSBs are unrelated to RAD51 phosphorylation by CHK1 or RAD51 function

In yeast, replisome stabilization requires the functional homolog of the human CHK1, Rad53. In the absence of Rad53, stalled replisomes collapse and replication intermediates become vulnerable to degradation by exonucleases and endonucleases [5], [6]. Thus, CHK1 inactivation may be instrumental for replication fork collapse also in humans. How CHK1 may contribute to stalled fork stabilization remains enigmatic, however, the main human recombinase RAD51 might have a pivotal and early role in this process [22], [42]. Since CHK1 phosphorylates RAD51 at T309 [17], DSBs generated upon replication stress induced by UCN-01 treatment could stem from loss of RAD51 function. Our results indicate that, even though RAD51 depletion results in formation of DSBs in unperturbed cells [43], these are MUS81-independent. This suggests that CHK1-dependent protection of perturbed forks from DSBs is unrelated to RAD51 phosphorylation. Indeed, neither RAD51 depletion nor the expression of an unphosphorylatable RAD51-T309A mutant, is sufficient to induce MUS81-dependent DSBs. Moreover, it is unlikely that cleavage by MUS81 is a consequence of combined loss of CHK1-dependent fork protection and CHK1-regulated RAD51 loading at distressed forks. Indeed, over-expression of a phosphomimetic RAD51-T309D mutant is not sufficient to revert MUS81-dependent DSBs after treatment with UCN-01 (our unpublished results). Alternatively, CHK1 might actively prevents targeting of collapsed forks by MUS81, as reported in fission yeast [44]. However, we detected no signs of a CHK1-dependent phosphorylation of MUS81 that can be reduced upon replication arrest by CHK1 inhibition. Further experiments are needed to clarify this point, which is outside the scope of this work.

### RAD52 is required for DSBs formation at perturbed forks *in vivo* and to stimulate MUS81 cleavage of a D-loop *in vitro*

**Figure 8:**
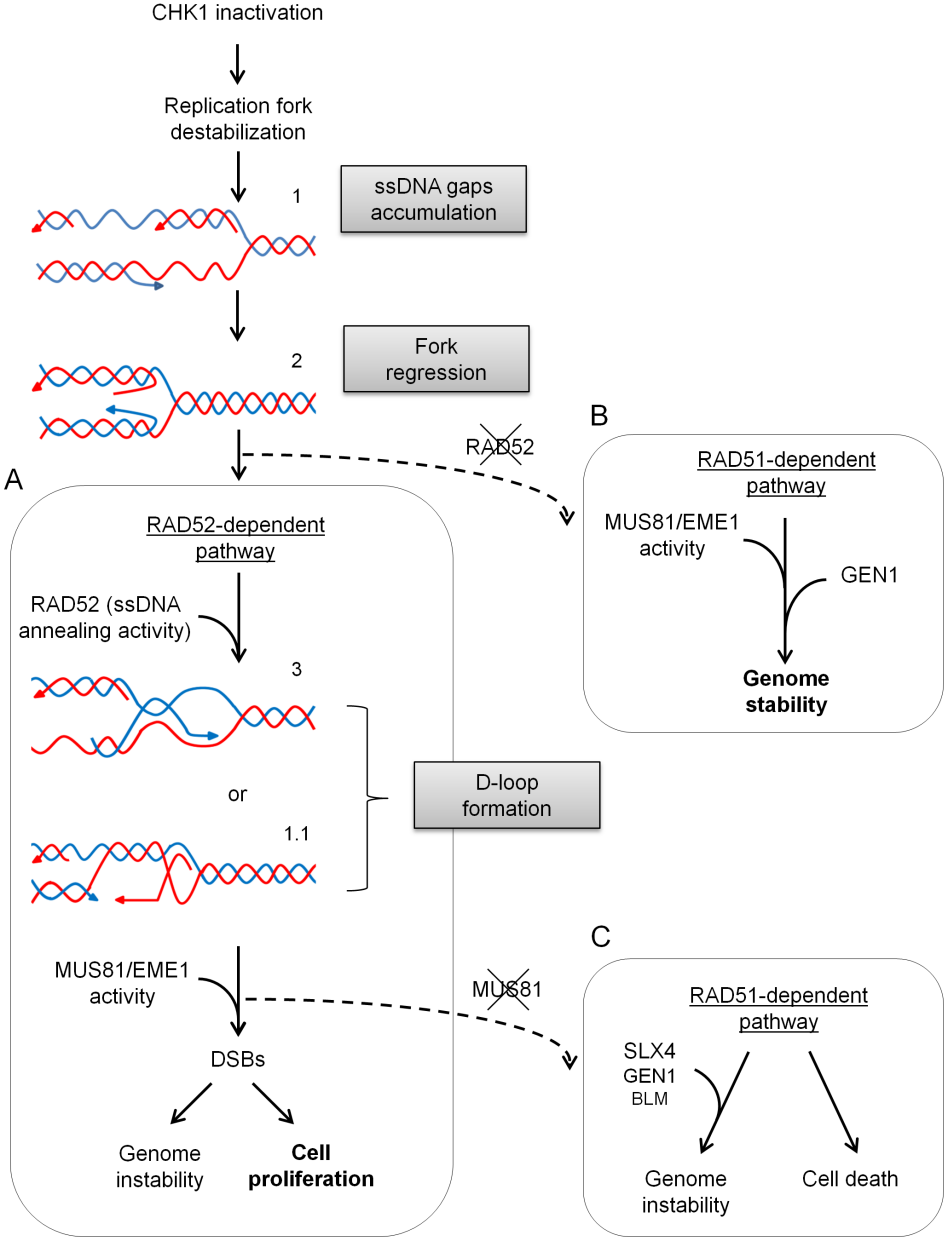
Model for processing of stalled forks in replication checkpoint-deficient cells. Inactivation of CHK1 determines destabilisation of stalled replication forks and accumulation of ssDNA gaps, likely at both the leading and the lagging strand. Stalled forks with ssDNA gaps (1) may undergo extensive extrusion of the newly-synthesized strands by fork regression (2) leading to a preferential engagement of RAD52 (A). RAD52, through its ssDNA annealing activity, would produce a D-loop intermediate (3) and possibly helps recruiting MUS81/EME1 complex by protein-protein interaction. Alternatively, RAD52 may assemble a D-loop intermediate from the ssDNA gap, either at the leading or the lagging strand behind the stalled fork (1.1). The D-loop intermediate is targeted by MUS81 resulting in DSBs and fork collapse. The BIR event that follows may involve subsequent requirement for viability of another SSE, GEN1. In the absence of a functional checkpoint (i.e. inactive CHK1), the RAD52-dependent pathway is a favourite, but inefficient, way of ensuring proliferation at the expense of genome stability. In the absence of RAD52, a RAD51-dependent mechanism (B) may be forcedly engaged. Viability of RAD52-deficient cells would require MUS81 and GEN1 to process the branched intermediates generated. This latter option, would limit genome instability at the cost of reduced survival, and would result in excessive lethality if MUS81 is also depleted. In contrast, MUS81 down-regulation, would stimulate a RAD51-mediated mechanism (C), but at the expense of both reduced cell viability and genome stability. Further details are discussed in the text.

It is generally thought that MUS81 may cleave RAD51-dependent recombination intermediates, such as HJs, mainly outside DNA replication. The identity of the MUS81 substrate and how it is generated at perturbed forks, however, remains unresolved [10], [12], [14], [45], [46], [47], [48]. Our data suggest that, upon fork collapse, MUS81 does not target a RAD51-dependent recombination intermediate, as similarly reported in WRN-deficient cells [21]. In fact, RAD51 depletion does not stimulate or revert formation of MUS81-dependent DSBs upon CHK1 inhibition, suggesting that MUS81 either targets the stalled forks directly, or processes other intermediates that form independently of RAD51. Since we observe that CHK1 inhibition and HU treatment stimulate RAD52 binding to chromatin, and that RAD52 depletion abrogates MUS81-dependent DSBs, we conclude that MUS81 does not cleave collapsed forks directly, but rather after the formation of a RAD52-dependent intermediate. It is worth noting that remodelling of collapsed forks prior to MUS81-dependent cleavage, might explain why DSBs are not formed immediately after replication arrest. Interestingly, MUS81-dependent DSBs accumulate after that CHK1 inhibition has induced a large amount of ssDNA, suggesting that MUS81 is cleaving an intermediate assembled from unreplicated leading or lagging strand. One of the substrate that could be generated by the ssDNA annealing activity of RAD52, perhaps through assistance of an helicase, is a D-loop, which is an ideal substrate for MUS81. Indeed, our *in vitro* studies support this hypothesis, and also demonstrate that MUS81 specifically targets D-loops assembled by RAD52. The apparent inability of MUS81/EME1 to cleave D-loops produced by RAD51, provides a mechanistic understanding of the RAD51 independency showed by DSBs formed by MUS81 *in vivo*. This conclusion is further reinforced by data in yeast, showing that MUS81 may act on RAD52-dependent D-loops produced at collapsed forks [12]. Such a D-loop might result from either pairing of the extruded leading or lagging strand after fork regression, or by the attempt to repair a ssDNA gap behind the replication fork (see Figure 8).

Formation of D-loop by RAD52 requires its ssDNA annealing activity, probably associated with SUMO-conjugation [49]. Interestingly, we notice that CHK1 inhibition determines a striking accumulation of high-molecular-weight forms of RAD52, which may correspond to SUMO-modification of RAD52 (Figure 3C). Thus, it is likely that replication distresses, induced in CHK1-deficient cells, elevate DNA annealing activity of RAD52, which would correlate with D-loop formation. Alternatively, enhanced RAD52 SUMOylation could be a consequence of impaired RAD51 loading, secondary to CHK1 inhibition. Indeed, in yeast rad51 mutants, increased SUMOylated Rad52 has been found [50]. However, our observations that chromatin loading of RAD51 is not reduced in CHK1-inhibited cells, and that RAD51 down-regulation does not result in MUS81-dependent DSBs, favour the first hypothesis. DSBs produced at distressed replication forks may be channelled to the BIR pathway, which can occur in a way dependent on RAD51 or RAD52 [51], [52]. Interestingly, inhibition of RAD51-dependent strand-invasion, during recovery from the combined UCN-01 and HU treatment, does not affect the level of DSBs. Thus, it is likely that DSBs produced by MUS81 in response to CHK1 inhibition, triggers a RAD52-dependent BIR pathway. Since RAD51-dependent or independent BIR events are expected to produce different intermediates [51], it is not surprising that BLM, which processes double HJs, does not seem particularly important for viability under our experimental conditions.

Recent reports have evidenced that RAD52 might contribute to survival of BRCA2-deficient cells, promoting repair of DNA damage arising in cells with defective RAD51 loading [53]. Our data indicate that chemical inhibition of RAD51, or its improper loading as it may occurs in BRCA2-deficient cells, minimally affects formation of MUS81-dependent DSBs or results in excessive MUS81-dependent DSBs. The most likely explanation is that DNA transaction induced at stalled forks when CHK1 is inhibited is peculiar, and does not occur when RAD51 is not functional but CHK1 is still active. Even though our observations support a cooperation between RAD52 and MUS81 in response to replication stress, their synergistic effect on viability suggests that these proteins have also independent functions, consistently with the observed milder phenotype of RAD52 knock-down cells, respect to MUS81 or RAD51-depleted cells. Absence of MUS81-dependent DSBs in BRCA2-defective cells may be also related to the function of BRCA2 in protecting the stalled forks from MRE11-dependent degradation [54]. Accordingly, in the absence of BRCA2, MRE11-dependent degradation could prevent formation of the MUS81 substrate. Indeed, increased levels of ssDNA are detected in MUS81 knock-down cells, which may be related to further exonucleolytic cleavage at distressed forks to favour a RAD51-dependent pathway.

### Loss of MUS81 or RAD52 in CHK1-deficient cells results in engagement of other resolvases that differently affect cell survival upon replication stress

We show that recovery from replication stress of MUS81-depleted cells requires a CHK1-regulated RAD51, a phenotype that we do not observe after RAD52 depletion. Strikingly, elevated RAD51 foci was reported in MUS81-null MEFs upon spontaneous fork collapse [25], and we show a reduced viability of MUS81-depleted cells after down-regulation of BLM, SLX4 or GEN1, all targeting uninterrupted branched intermediates, most likely generated downstream RAD51. From this point of view, it is possible that structures left unprocessed by MUS81 are then channelled back into a RAD51-dependent recombination to ensure viability, perhaps with the help of RAD52 and CHK1 (see Figure 8).

Also depletion of RAD52, preventing the formation of the MUS81 substrate, would channel collapsed forks to a RAD51 route, as suggested by the strong accumulation of RAD51 on chromatin. Most importantly, concomitant depletion of RAD52 and MUS81 gives a similar increase in the amount of chromatin-bound RAD51, but also results in a strong reduction of viability. Interestingly, poor viability of RAD52/MUS81 depleted cells after checkpoint inactivation is ameliorated by RAD51-depletion. Similarly, viability of the RAD52/MUS81-depleted cells is improved by knock-down of GEN1, which is responsible for the DSBs observed in this background. Thus, it is likely that loss of RAD52, precluding the formation of the MUS81 substrate, determines the formation of a RAD51-dependent intermediate that should be normally processed by MUS81, which is also absent, and becomes toxic after GEN1 cleavage. Since in yeast Yen1(GEN1) may substitute for Mus81 during repair of DSBs at perturbed forks [55], our results might suggest that such relationship is not valid in human cells. However, if RAD52 is present, such “redundancy” between MUS81 and GEN1 can be observed also in our hands (see Figure S9). Thus, it is likely that intermediates accumulating downstream of RAD51 become toxic once they are cleaved by GEN1, because of the absence of RAD52 or of loss of CHK1-mediated regulation. Given the well-known difference in substrate specificity between MUS81 and GEN1, with the latter preferentially acting on the uninterrupted intermediates [56], it is possible that these types of structures (i.e. single HJ or reversed forks) are accumulating in the RAD52/MUS81 double-depleted cells.

### The RAD52/MUS81-dependent pathway is responsible for reduced fork recovery and increased chromosome instability associated to CHK1 inhibition

Even though MUS81 is important to limit cell death, its depletion minimally affects chromosomal damage in checkpoint-deficient cells (Figure 7). However, depletion of MUS81 rescues the instability occurring at common fragile sites after oncogene expression or under unperturbed conditions [18], [41], [57]. In contrast, depletion of RAD52 reduces genome instability in the absence of CHK1. Since RAD52 down-regulation is expected to prevent formation of the MUS81 substrate, it is tempting to speculate that genome instability occurring upon checkpoint failure mostly depends on MUS81 cleavage, or further processing of the uncleaved substrate (see Figure 8). Depletion of RAD52 also rescues the ability of stalled forks to restart under CHK1 inhibition. This might be related to a forced switch to the RAD51-dependent BIR pathway that is expected to be faster than that, RAD51-independent, engaged in wild-type cells, as proposed in yeast [58], [59]. Interestingly, depletion of RAD52, almost specifically, improves the ability of stalled forks to restart following CHK1 inhibition, i.e. after checkpoint inactivation. This observation, together with the evidence that, in our experimental condition, CHK1-inhibited cells are blocked in S-phase, might suggest that preferential engagement of a RAD52-dependent pathway is linked to an attempt of cells to activate a checkpoint response bypassing CHK1 inhibition, as speculated for BIR in yeast [58]. Our observation that RAD52 depletion also results in more *de novo* origin firing, is consistent with a “checkpoint-like” function of the RAD52-MUS81 pathway, however, additional studies are necessary to confirm such intriguing hypothesis.

Altogether, our results show that MUS81 is responsible for the generation of DSBs after replication stress induced when CHK1 activity is impaired. They also demonstrate that the generation of MUS81-dependent DSBs is not the consequence of an altered function of RAD51, but depends on the presence of RAD52. Furthermore, integrity of this RAD52/MUS81-dependent mechanism is critical for cell viability under replication stress, and its loss engages toxic RAD51-dependent transactions. Given that replication stress has been associated with cancer progression, and since CHK1 inhibitors are considered in anticancer therapy, our findings may also improve an educated approach to replication checkpoint inhibition in cancer cells, by capitalizing on potential synergistic effects and reduced functionality of the recombination factors. It is clear, for example, that inhibition of RAD52 in CHK1-deficient cells should reduce the viability of cancerous cells, and should prevent genetic instability and thereby the risk of resistance emergence.

## Materials and Methods

### Cell cultures

The GM01604 hTERT-immortalised normal human fibroblasts, the 293T cells and the WI-38 SV40-transformed normal human fibroblasts were obtained from Coriell Cell Repositories (Camden, NJ, USA) or American Type Culture Collection (Manassas, VA, USA). Cells were cultured in Dulbecco's modified Eagle's medium (DMEM; Life Technologies) supplemented with 15% FBS (Boehringer Mannheim) for the GM01604 fibroblasts and 10% FBS for WI-38 fibroblasts and 293T cells. All the cells were incubated at 37°C in a 5% CO_2_ atmosphere.

### Chemicals

HU and BrdU were obtained from Sigma-Aldrich. HU was dissolved in sterile PBS as a stock solution (200 mM) and stored at +4°C.

BrdU was dissolved in sterile PBS as a stock solution (3 mg/ml) and stored at −20°C. UCN-01 (Alexis Biochemicals) was used at 400 nM concentration to inhibit CHK1 activity, while to inhibit ATR activity the ETP-46464 compound (a gift of Dr. Fernandez-Capetillo) was used at 10 µM concentration. The specific RAD51 inhibitor B02 was from Merck chemicals, and was used at 27 µM according to Huang et al [60].

### RNA interference and genetic complementation experiments

MUS81, CHK1, CHK2, ATR, RAD9, TOPBP1, TIPIN, RAD51, BLM, SLX4, GEN1 and RAD52 expression were knocked down by transfection with SMARTpool siRNAs (Dharmacon) directed against proteins of interest at the final concentration of 10 nM. Transfection was performed using Interferin (Polyplus) according to the manufacturer's instructions. As a control, a siRNA duplex directed against GFP was used.

For genetic complementation experiments, cells were first transfected with a mix of two distinct siRNA oligos targeting the UTR region of human RAD51 (Qiagen Flexi tube; cat# SI00045010 and SI02629837) at a 10 nM concentration using HiPerfect reagent (Qiagen) and then nucleofected using the Amaxa device (Kit #VACA-01) to express RNAi-resistant wild-type (RAD51wt), phosphorylation-defective (RAD51-T309A), or a phosphomimetic (RAD51-T309D) mutant form of RAD51 (see Text S1).

Nucleofection of plasmids a was performed using 2 µg of supercoiled DNA according to the manufacturer's instructions.

### Western blot and chromatin fractionation

Western blot and chromatin fractionation were performed as described in Franchitto et al [14]. Blots were incubated with primary antibodies against: MUS81 (Abcam), SLX4 (Abcam), Phospho-Ser345-CHK1 (Cell Signaling), CHK1 (Santa Cruz Biotechnology), BLM (Santa Cruz Biotechnology), CHK2 (Calbiochem), ATR (Calbiochem), RAD9 (Calbiochem), TOPBP1 (Bethyl), TIPIN (Bethyl), RAD51 (Abcam) and RAD52 (Santa Cruz Biotechnology), PCNA (Santa Cruz Biotechnology), Tubulin β (Sigma-Aldrich), phoshpo-H3 histone (Santa Cruz Biotechnology) and Lamin B1 (Abcam). The anti-GEN1 antibody was a kind gift of Prof. Yungui Yang (Beijing Genomics Institute). After incubations with horseradish peroxidase-linked secondary antibodies (Vector Laboratories), the blots were developed using the chemiluminescence detection kit ECL-Plus (Amersham) according to the manufacturer's instructions.

### Immunopurification of human MUS81/EME1 complex

To immunopurify the human MUS81/EME1 complex, 293T cells were transiently transfected with a 1∶1 ratio of plasmids expressing the Myc-tagged MUS81 and the GST-tagged EME1 ORFs. Transfection was performed using the Dreamfect reagent (Ozbiosciences) according to the manufacturer's direction. Cells were collected 60 h after transfection and nuclear pellets stored frozen for subsequent immunopurification. For immunopurification, nuclei obtained from 5×10^7^ cells were lysed in CSK buffer (200 mM NaCl, 300 mM sucrose, 3 mM MgCl2, 1 mM DTT, 10 mM PIPES - pH 6.8) containing 0.5% Triton X-100, protease inhibitors and benzonase. After removal of nuclear debris by centrifugation, cleared lysate was incubated with 0.3 ml of agarose beads conjugated with anti-Myc antibodies under rotation. After incubation, the beads were extensively washed with TNT buffer (50 mM Tris/Cl buffer pH 7.6, containing 300 mM NaCl, 0.5% Triton X-100, 2 mM DTT, 3 mM MgCl2 and protease inhibitors). After washing, beads were incubated under rotation with the Myc peptide to elute the MUS81/EME1 complex. One tenth of the eluate was analysed for the presence of the MUS81/EME1 complex using Coomassie staining and the remaining volume of eluate was further purified by incubation with GSH-agarose to capture the intact MUS81/EME1 heterodimer. Finally MUS81/EME1 heterodimer was retrieved by elution using PBS containing 25 mM Glutathione, concentrated and the amount and purity of the complex estimated by SDS-PAGE followed by Coomassie staining.

### Comet assay

The occurrence of DNA double-strand breaks was evaluated by neutral Comet assay as described [61]. Alternatively, cells were subjected to Comet assay under alkaline conditions to detect both DSBs and single-stranded DNA gaps or nicks. Cell DNA was stained with ethidium bromide (Sigma) and examined at 40× magnification with an Olympus fluorescence microscope. Slides were analyzed by a computerized image analysis system (Comet IV, Perceptive UK). To assess the amount of DNA damage, computer-generated tail moment values (tail length×fraction of total DNA in the tail) were used. A minimum of 200 cells was analysed for each experimental point. Apoptotic cells (smaller comet head and extremely larger comet tail) were excluded from the analysis to avoid artificial enhancement of the tail moment.

### D-loop cleavage assay

To determine whether RAD52 and RAD51 produce the MUS81/EME1 cleavable structures we generated the D-Loops produced by these proteins. Human RAD51 and RAD52 proteins were purified as described in [62], [63], [64]. The concentration of the proteins were determined using their molar extinction coefficients; 12,800M^−1^cm^−1^ (RAD51) and 40,380 M^−1^cm^−1^ (RAD52). RAD51- or RAD52-mediated D-loops were produces essentially as described in [65]. Briefly, 20 nM (molecules) γ-P^32^-labeled ssDNA oligonucleotide (5′-ATT TTG TTC ATG GTA GAG ATT CTC TTG TTG ACA TTT TAA AAG AGC GTG G-3′) was incubated with 1 µM RAD52 or RAD51 protein at 37°C for 7 minutes in the reaction buffer (50 mM HEPES-NaOH (pH 7.5), 5 mM MgCl_2_, 100 mM NaCl_2_, 0.1 mg/ml BSA and 1 mM DTT). The RAD51 reaction also contained 1 mM ATP. D-loop formation was initiated by addition of 10 nM (molecules) of φX174 RFI supercoiled dsDNA followed by incubation at 37°C for 20 min. The protein-free D-loops were produced by mixing the oligonucleoted with the supercoiled dsDNA in the reaction buffer, heating the mixture to 95°C and then slowly cooling the mixture to room temperature. The MUS81/EME1 complex was then added to the D-loops at the indicated concentrations and the reactions were further incubated for 20 or 60 min at 37°C. The reaction was stopped by adding 1 µl of 10% SDS, followed by immediately adding 1 µl of 10 mg/ml proteinase K and incubation at 37°C for 30 min. 6X DNA Gel Loading Dye (Thermo) was added to the reaction and the samples were resolved on the 0.8% agarose gel in 1X TAE Buffer at 5 V/cm at room temperature. The reaction products were visualized using a phosphorimager system (GE Healthcare). D-loops were quantified using ImageJ software [66].

### 3′-flap cleavage assay

Substrates representing 3′-flap (JLBD20) and nicked product (LMBD20) were produced by annealing the following oligonucleotides: (GGATGGCTTAGAGCTT AATTCCGCTCATGGATGCTATCACGC), L (CGTACTGCAATCTTGAACCG-Cy5-GGAA TTAAGCTCTAAGCCATCC), M (GGATGGCTTAGAGCTTAATTCC) and BD20 (CGGTTC AAGATTGCAGTACG, by incubating the Cy5-labeled oligo L with 1.5-fold excess of the two unlabeled nucleotides in T50 buffer (Tris-HCl pH 7.5, 50 mM NaCl) at 95°C and gradually cooling to 25°C in a dry bath over 4 hours. The substrate is one of those preferred by MUS81 as described in Ciccia and colleagues [8]. The reactions contained nuclease buffer (50 mM Tris-HCl pH 7.5, 100 mM NaCl, 5 mM MgCl2, 20 mM Glycine, 2 mM Dithiothreitol),10 nM JLBD20 oligo in reaction buffer, and 100 nM of MUS81-EME1, RAD52 or both proteins. The reaction mixtures were incubated at 37°C for 90 minutes and stopped by adding 0.5% SDS, followed by immediately adding 0.3 mg/ml proteinase K and incubation at 37°C for 30 minutes. The samples were then separated on a 15% non-denaturing TBE-PAGE gel and analyzed with Cy5 detection using the BioRad Chemidoc system. The percentage of nicked product was quantified using ImageJ software.

### Pull-down experiments

To determine whether RAD52 associates with MUS81 we performed pull-down experiments using purified His-tagged RAD52 (see above) as bait and HeLa nuclear extract (NE) as source of the pray. Briefly, 5 µg of recombinant RAD52 was incubated overnight with 1 mg of NE in binding buffer (Tris/Cl buffer pH 7.6 containing 150 mM NaCl and 0.5% Triton X-100). One-fiftieth of the NE was put apart to be used as input.

The pull-down material was then incubated for 1 h at RT with 4 µg of anti-His antibody-coupled Protein G to capture RAD52 complexes, and after extensive washing in binding buffer, proteins were released by incubation in 1× Laemmli sample buffer.

### Immunofluorescence staining of replication foci using CldU and IdU

DNA replication sites were visualized by incorporation of chlorodeoxyuridine (CldU) and iododeoxyuridine (IdU) into DNA. GM01604 cells were transfected with siRNAs directed against GFP (control) or against MUS81 or RAD52, and 48 h thereafter treated for 6 h with 400 nM UCN-01 alone or in combination with 2 mM HU. The CldU label (25 µM) was added 10 min before treatments and after 6 h cells were washed extensively and labelled with 200 µM IdU for 45 min. Cells were then washed with PBS, fixed with cold 70% ethanol, and stored at 4°C. Antibody staining was performed as previously reported [18]. Images were acquired as greyscale files using Metaview software (MDS Analytical Technologies) and processed using Adobe Photoshop CS3 (Adobe). For each time point, at least 200 nuclei were examined by two independent investigators and foci were scored at 60×.

### LIVE/DEAD staining

GM01604 cells were transfected with siRNAs directed against GFP (control), or against MUS81, CHK1, ATR, TIPIN, RAD51, SLX4, BLM, GEN1 and RAD52 (Qiagen).

Viability was evaluated by the LIVE/DEAD assay (Sigma-Aldrich) according to the manufacturer's instructions. Cell number was counted in randomly chosen fields and expressed as percent of dead cells (number of red nuclear stained cells/total cell number). For each time point, at least 200 cells were counted.

### Chromosome preparations and analysis

WI-38 SV40-transformed fibroblasts were transfected with siRNAs directed against GFP (siCtrl), MUS81 (siMUS81) or RAD52 (siRAD52). Forty-eight hours after interference, cells were treated for 6 h with 2 mM HU or pre-treated for 1 h with 400 nM UCN-01 and then 6 h together with HU. At the end of treatments, all the cells were recovered in drug-free medium for 21 h. Cell cultures were incubated with colcemid (0.2 µg/ml) at 37°C for 3 h until harvesting. Cells for metaphase preparations were collected and prepared as previously reported [67]. The analysis of chromosomal aberrations was performed by scoring at least 100 Giemsa-stained metaphases per experimental point.

## Supporting Information

Figure S1 Analysis of DSBs by γH2AX immunofluorescence. (**A**) DSBs accumulation was assessed by γH2AX immunofluorescence in GM01604 cells transfected with ATR or CHK1 siRNA alone or in combination with MUS81 siRNA. Cells were treated with 2 mM HU for 6 h before subjecting to IF and scored for the presence of pan-nuclear γH2AX staining. Graph shows data presented as mean of the % of positive nuclei +/− SE. In the panel (**B**) representative images from selected samples are shown.
(PDF)

Figure S2 MUS81 down-regulation suppresses DSBs formation in S-phase cells. (**A**) Analysis of cell cycle progression. GM01604 cells were synchronized as described in Text S1 and transfected with siRNAs directed against GFP (siCtrl) or MUS81 (siMUS81). Forty-eight hours after interference, cells were treated with 400 nM CHK1 inhibitor (UCN-01) for 1 h and then with 2 mM HU for 6 h. At the end of the treatment, cells were collected and subjected to FACS analysis as described in Text S1. (**B**) Evaluation of DSBs accumulation after replication arrest. GM01604 cells were synchronized and treated as in (A) and then subjected to neutral comet assay. Data are presented as fold increase respect to the untreated, siCtrl-transfected control. Error bars represent standard errors.
(PDF)

Figure S3 MUS81 down-regulation does not alter cell cycle arrest of progression of checkpoint-deficient cells. (**A**) Measurement of percentage of S-phase cells. GM01604 cells were transfected with control siRNAs (siCtrl) or siMUS81. Forty-eight hours later, cells were treated with UCN-01 or ETP-46464 for 1 h and then exposed overnight with 2 mM HU. After HU-treatment, cells were pulse-labeled with 30 mM BrdU for 30 min and collected at the indicated recovery times to be subjected to immunofluorescence analysis as in Text S1. Replicating DNA was visualized using anti-BrdU antibody. In the graph data are presented as percentage of BrdU-positive cells and are mean of three independent experiments. Error bars represent standard error. Where not depicted, standard errors were <15% of the mean. (**B**) Analysis of MUS81 down-regulation in synchronized cells. GM01604 cells were synchronized as described in Materials and Methods” and transfected with control siRNAs (siCtrl) or siMUS81. Forty-eight hours after interference, cells were treated with UCN-01 for 1 h and then with HU for 6 h. Samples were collected and subjected to immunoblotting analysis at the indicated times to assess MUS81 interference at the beginning of HU-treatment (48 h after interference) and at the end of recovery period (72 h after interference). Depletion of MUS81 was verified using the anti-MUS81 antibody. PCNA was used as loading control. (**C**) Analysis of cell cycle progression after replication arrest. GM01604 cells synchronized and treated as in (B), were subjected to FACS analysis.
(PDF)

Figure S4 Analysis of the formation of DSBs or ssDNA gaps, nicks and DSBs at different time points after checkpoint inhibition. (**A**) GM01604 cells were treated as indicated and analyzed for the presence of DSBs by neutral comet assay at different time-points. Data are presented as fold increase of tail moment and are mean of three independent experiments. Error bars represent standard errors. (**B**) GM01604 cells were treated as indicated and analyzed for the presence of DSBs by neutral comet assay at different time-points. Data are presented as fold increase of tail moment and are mean of three independent experiments. Error bars represent standard errors. (**C**) GM01604 cells were treated as indicated and analyzed for the presence of ssDNA gaps, nicks and DSBs by alkaline comet assay at different time-points. Data are presented as fold increase of tail moment and are mean of three independent experiments. Error bars represent standard errors. Treatments were: 2 mM HU alone or in combination with 400 nM UCN-01.
(PDF)

Figure S5 Analysis of RAD51 relocalization in foci in the absence of TIPIN. GM01604 cells were transfected with control siRNAs (siCtrl), siCHK1 or siTIPIN. Cells treated with UCN-01 for 1 h were used as control. Forty-eight hours after RNAi or treatment with CHK1 inhibitor, cells were treated with 2 mM HU for 6 h and then subjected to RAD51 immunofluorescence analysis. Cells were stained with an antibody against RAD51. Graph shows quantification of the percentage of RAD51-positive nuclei for each experimental condition. Data are presented as fold increase respect to the control. Error bars represent standard error. Where not depicted, standard errors were <15% of the mean. In the panel representative images from the HU-treated samples are shown.
(PDF)

Figure S6 Analysis of the formation of DSBs in BRCA2-mutant lymphoblasts. HSC-62 lymphoblastoid cells (a gift of Dr. Rosselli, CNRS) were treated with 2 mM HU alone or in combination with 400 nM UCN-01 as indicated and analyzed for the presence of DSBs by neutral comet assay after 6 h from treatment. Data are presented as fold increase of tail moment and are mean of three independent experiments. Error bars represent standard errors.
(PDF)

Figure S7 Analysis of the formation of ssDNA gaps, nicks and DSBs after down-regulation of different recombination factors. GM01604 cells were transfected with control siRNAs directed against GFP (siCtrl) or MUS81 (siMUS81), RAD52 (siRAD52), RAD51 (siRAD51) and a combination of MUS81 and RAD52 (siMUS81/RAD52) and treated with UCN-01 or 2 mM HU alone, or in a combination of both treatments for 6 h before alkaline comet assay. Data are presented as fold increase of tail moment and are mean of three independent experiments. Error bars represent standard errors.
(PDF)

Figure S8 Effect of RAD51 inhibition on DSBs formation in RAD52/MUS81 double-depleted cells. GM01604 cells were transfected with control siRNAs directed against GFP (siCtrl) or a combination of MUS81 and RAD52 siRNA (siMUS81/RAD52) and treated 2 mM HU alone or in combination with 400 nM UCN-01 for 6 h, with or without the RAD51 inhibitor. Then, cells were washed and recovered for 4 h before neutral comet assay. Graph shows data presented as mean tail moment +/− SE from three independent experiments.
(PDF)

Figure S9 Viability of cells depleted of different SSEs or BLM after replication stress induced by CHK1 inhibition. (**A**) GM01604 cells were transfected with siRNAs directed against GFP (siCtrl) or against the indicated SSEs or BLM. (**B**) Evaluation of cell death in cells were transfected with a combination of the indicated RNAi oligos. In all cases, cells were treated 48 h post-transfection with 400 nM UCN-01 alone or in combination with 2 mM HU for 6 h, followed by recovery for 18 h in drug-free medium prior to evaluation of cell death by the LIVE/DEAD assay. Graph shows data presented as means +/− SE from three independent experiments. Western blot panels show actual depletion levels obtained for each of the depletion analyzed in (A) and (B).
(PDF)

Figure S10 Viability of cells depleted of RAD52 in combination with different SSEs or BLM (**A**) GM01604 cells were transfected with siRNAs directed against RAD52 and MUS81, alone or in combination with RNAi oligos against the SSEs SLX4 and GEN1 (**B**) Evaluation of cell death in cells were transfected with a combination of the indicated RNAi oligos. In all cases, cells were treated 48 h post-transfection with 400 nM UCN-01 alone or in combination with 2 mM HU for 6 h, followed by recovery for 18 h in drug-free medium prior to evaluation of cell death by the LIVE/DEAD assay. Graph shows data presented as means +/− SE from three independent experiments. Western blot panels show actual depletion levels obtained for each of the depletion analyzed (B); For what concerns efficiency of the oligos used for multiple depletion analyzed in panel (A), refer to Figure 3D.
(PDF)

Text S1 The file contains supplementary methods for immunofluorescence; evaluation of S-phase content, flow-cytometry analysis, and site-directed mutagenesis.
(DOC)
