# Supplementary figures and images for: Survival of the Replication Checkpoint Deficient Cells Requires MUS81-RAD52 Function
Source: PLoS Genet. 2013 Oct 31;9(10):e1003910. doi: 10.1371/journal.pgen.1003910 (PMC3814295; doi:10.1371/journal.pgen.1003910)

A

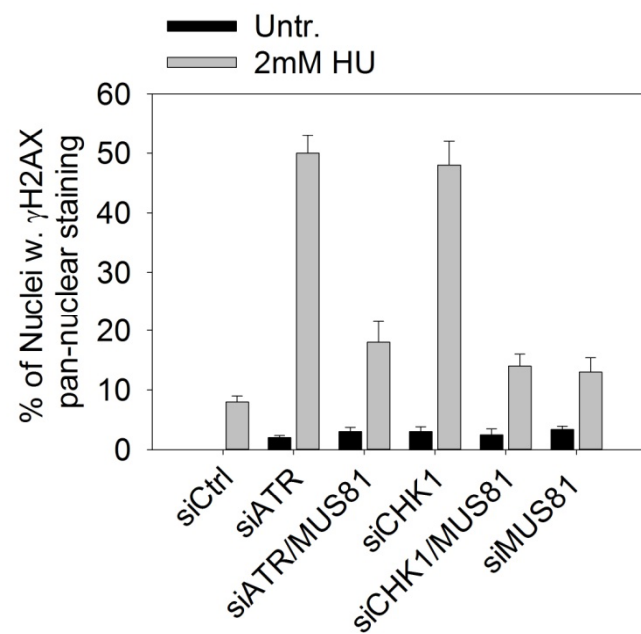

B

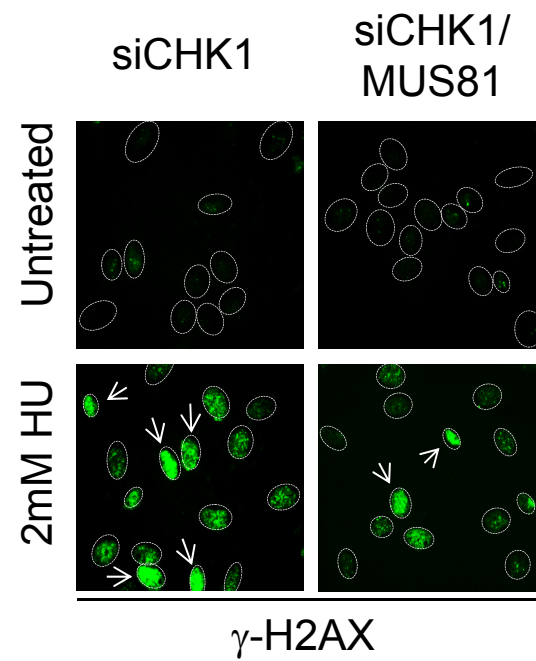

Figure S1

Supplement: Figure S1 — Analysis of DSBs by γH2AX immunofluorescence. (A) DSBs accumulation was assessed by γH2AX immunofluorescence in GM01604 cells transfected with ATR or CHK1 siRNA alone or in combination with MUS81 siRNA. Cells were treated with 2 mM HU for 6 h before subjecting to IF and scored for the presence of pan-nuclear γH2AX staining. Graph shows data presented as mean of the % of positive nuclei +/− SE. In the panel (B) representative images from selected samples are shown. (PDF) [file pgen.1003910.s001.pdf]

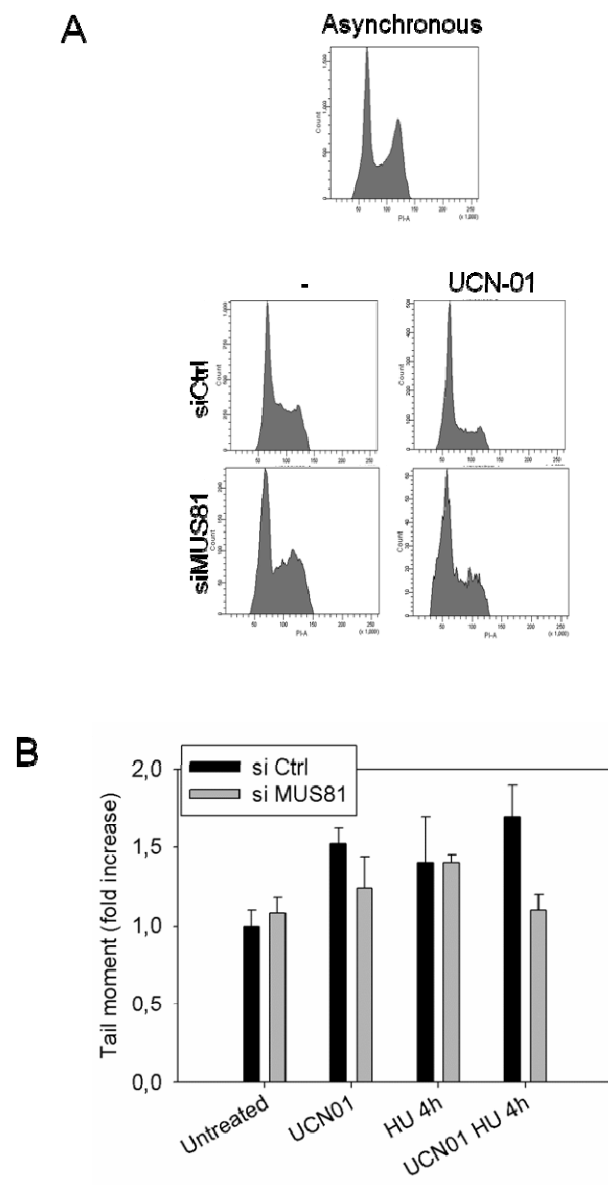

Figure S2

Supplement: Figure S2 — MUS81 down-regulation suppresses DSBs formation in S-phase cells. (A) Analysis of cell cycle progression. GM01604 cells were synchronized as described in Text S1 and transfected with siRNAs directed against GFP (siCtrl) or MUS81 (siMUS81). Forty-eight hours after interference, cells were treated with 400 nM CHK1 inhibitor (UCN-01) for 1 h and then with 2 mM HU for 6 h. At the end of the treatment, cells were collected and subjected to FACS analysis as described in Text S1. (B) Evaluation of DSBs accumulation after replication arrest. GM01604 cells were synchronized and treated as in (A) and then subjected to neutral comet assay. Data are presented as fold increase respect to the untreated, siCtrl-transfected control. Error bars represent standard errors. (PDF) [file pgen.1003910.s002.pdf]

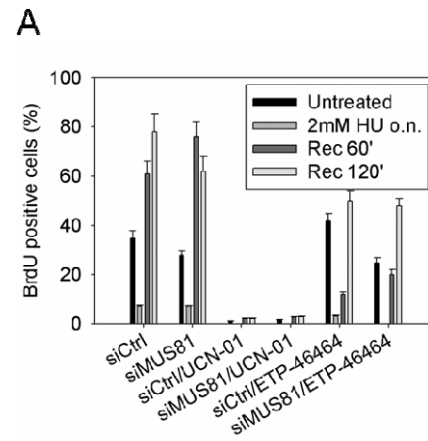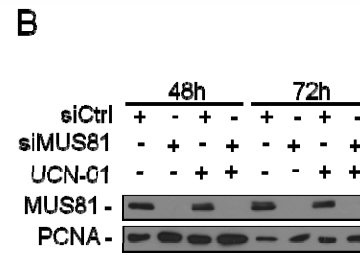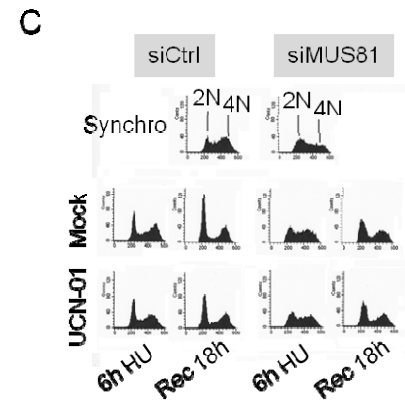

Figure S3

Supplement: Figure S3 — MUS81 down-regulation does not alter cell cycle arrest of progression of checkpoint-deficient cells. (A) Measurement of percentage of S-phase cells. GM01604 cells were transfected with control siRNAs (siCtrl) or siMUS81. Forty-eight hours later, cells were treated with UCN-01 or ETP-46464 for 1 h and then exposed overnight with 2 mM HU. After HU-treatment, cells were pulse-labeled with 30 mM BrdU for 30 min and collected at the indicated recovery times to be subjected to immunofluorescence analysis as in Text S1. Replicating DNA was visualized using anti-BrdU antibody. In the graph data are presented as percentage of BrdU-positive cells and are mean of three independent experiments. Error bars represent standard error. Where not depicted, standard errors were <15% of the mean. (B) Analysis of MUS81 down-regulation in synchronized cells. GM01604 cells were synchronized as described in Materials and Methods” and transfected with control siRNAs (siCtrl) or siMUS81. Forty-eight hours after interference, cells were treated with UCN-01 for 1 h and then with HU for 6 h. Samples were collected and subjected to immunoblotting analysis at the indicated times to assess MUS81 interference at the beginning of HU-treatment (48 h after interference) and at the end of recovery period (72 h after interference). Depletion of MUS81 was verified using the anti-MUS81 antibody. PCNA was used as loading control. (C) Analysis of cell cycle progression after replication arrest. GM01604 cells synchronized and treated as in (B), were subjected to FACS analysis. (PDF) [file pgen.1003910.s003.pdf]

A

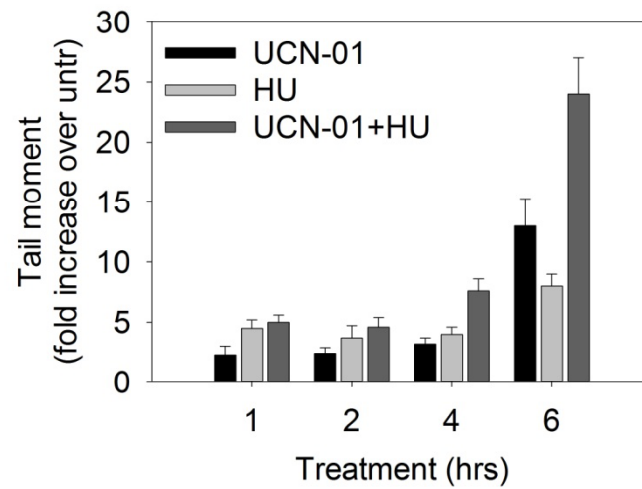

B

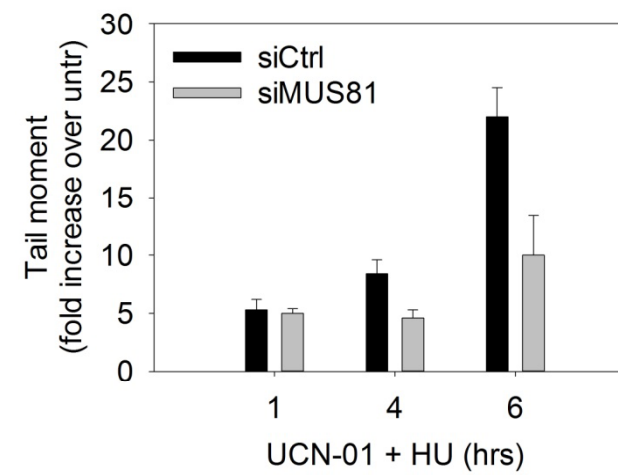

C

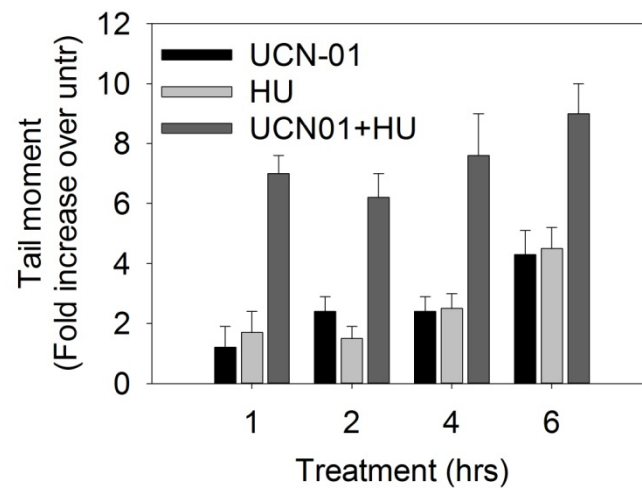

Figure S4

Supplement: Figure S4 — Analysis of the formation of DSBs or ssDNA gaps, nicks and DSBs at different time points after checkpoint inhibition. (A) GM01604 cells were treated as indicated and analyzed for the presence of DSBs by neutral comet assay at different time-points. Data are presented as fold increase of tail moment and are mean of three independent experiments. Error bars represent standard errors. (B) GM01604 cells were treated as indicated and analyzed for the presence of DSBs by neutral comet assay at different time-points. Data are presented as fold increase of tail moment and are mean of three independent experiments. Error bars represent standard errors. (C) GM01604 cells were treated as indicated and analyzed for the presence of ssDNA gaps, nicks and DSBs by alkaline comet assay at different time-points. Data are presented as fold increase of tail moment and are mean of three independent experiments. Error bars represent standard errors. Treatments were: 2 mM HU alone or in combination with 400 nM UCN-01. (PDF) [file pgen.1003910.s004.pdf]

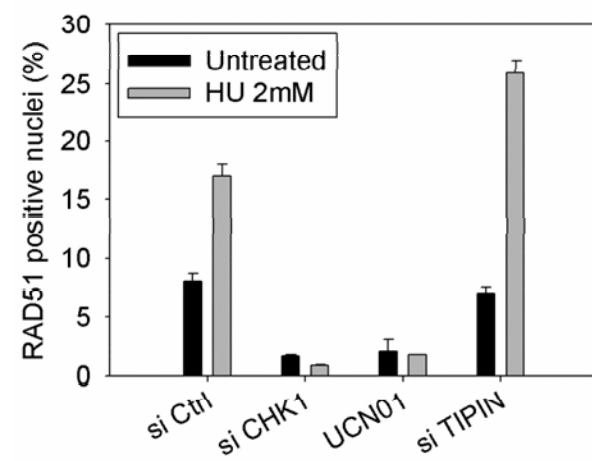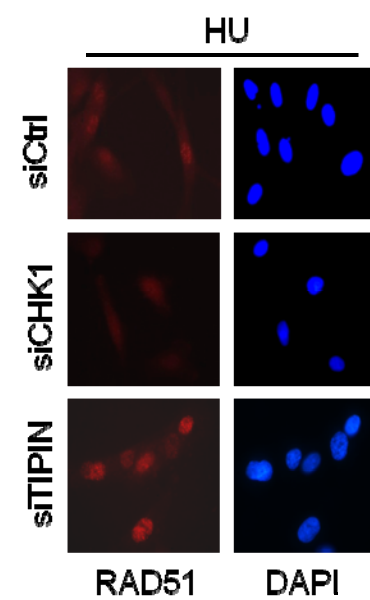

Figure S5

Supplement: Figure S5 — Analysis of RAD51 relocalization in foci in the absence of TIPIN. GM01604 cells were transfected with control siRNAs (siCtrl), siCHK1 or siTIPIN. Cells treated with UCN-01 for 1 h were used as control. Forty-eight hours after RNAi or treatment with CHK1 inhibitor, cells were treated with 2 mM HU for 6 h and then subjected to RAD51 immunofluorescence analysis. Cells were stained with an antibody against RAD51. Graph shows quantification of the percentage of RAD51-positive nuclei for each experimental condition. Data are presented as fold increase respect to the control. Error bars represent standard error. Where not depicted, standard errors were <15% of the mean. In the panel representative images from the HU-treated samples are shown. (PDF) [file pgen.1003910.s005.pdf]

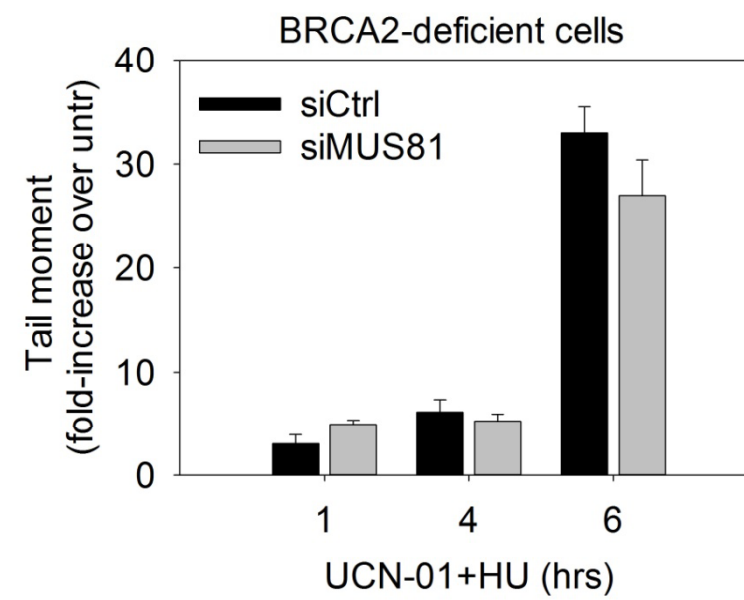

Figure S6

Supplement: Figure S6 — Analysis of the formation of DSBs in BRCA2-mutant lymphoblasts. HSC-62 lymphoblastoid cells (a gift of Dr. Rosselli, CNRS) were treated with 2 mM HU alone or in combination with 400 nM UCN-01 as indicated and analyzed for the presence of DSBs by neutral comet assay after 6 h from treatment. Data are presented as fold increase of tail moment and are mean of three independent experiments. Error bars represent standard errors. (PDF) [file pgen.1003910.s006.pdf]

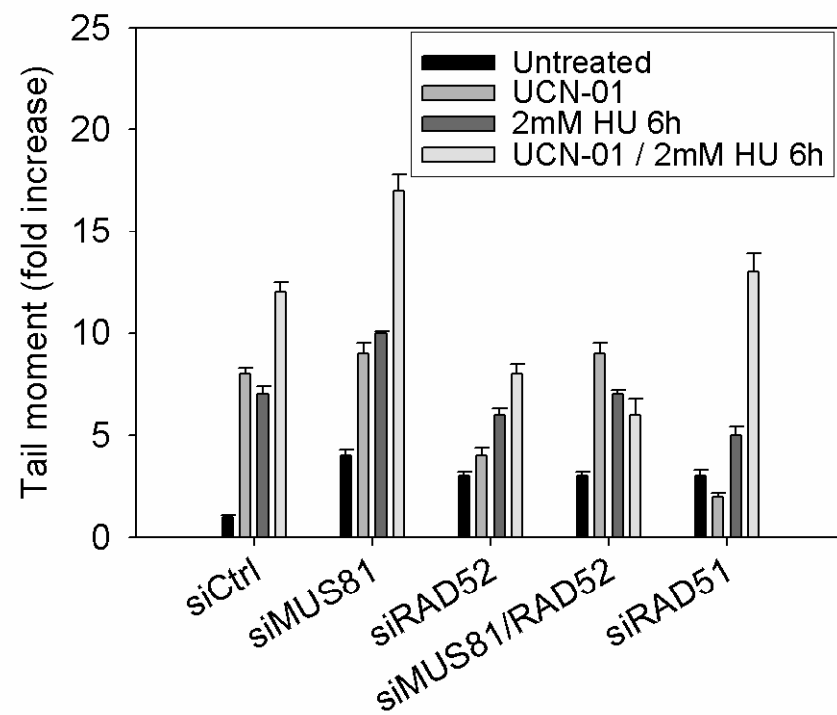

Figure S7

Supplement: Figure S7 — Analysis of the formation of ssDNA gaps, nicks and DSBs after down-regulation of different recombination factors. GM01604 cells were transfected with control siRNAs directed against GFP (siCtrl) or MUS81 (siMUS81), RAD52 (siRAD52), RAD51 (siRAD51) and a combination of MUS81 and RAD52 (siMUS81/RAD52) and treated with UCN-01 or 2 mM HU alone, or in a combination of both treatments for 6 h before alkaline comet assay. Data are presented as fold increase of tail moment and are mean of three independent experiments. Error bars represent standard errors. (PDF) [file pgen.1003910.s007.pdf]

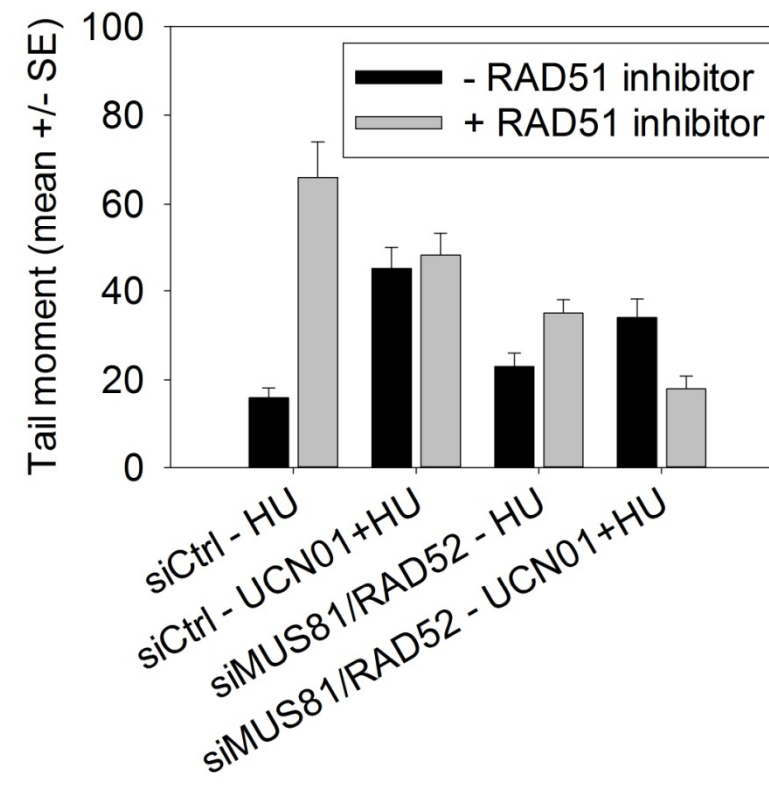

Figure S8

Supplement: Figure S8 — Effect of RAD51 inhibition on DSBs formation in RAD52/MUS81 double-depleted cells. GM01604 cells were transfected with control siRNAs directed against GFP (siCtrl) or a combination of MUS81 and RAD52 siRNA (siMUS81/RAD52) and treated 2 mM HU alone or in combination with 400 nM UCN-01 for 6 h, with or without the RAD51 inhibitor. Then, cells were washed and recovered for 4 h before neutral comet assay. Graph shows data presented as mean tail moment +/− SE from three independent experiments. (PDF) [file pgen.1003910.s008.pdf]

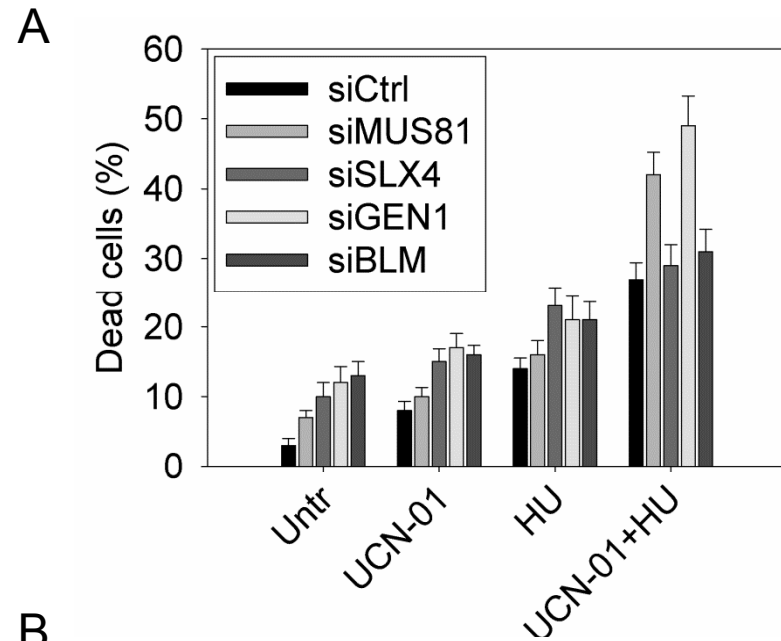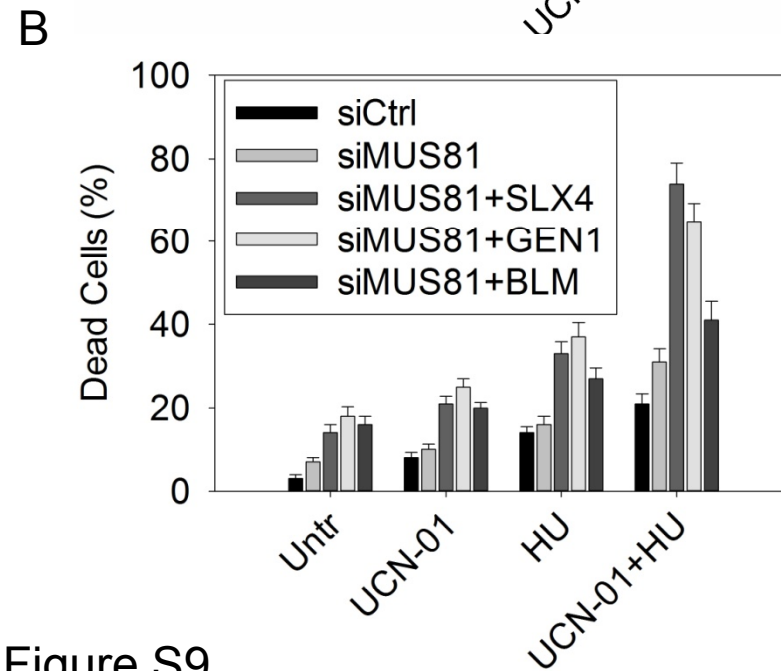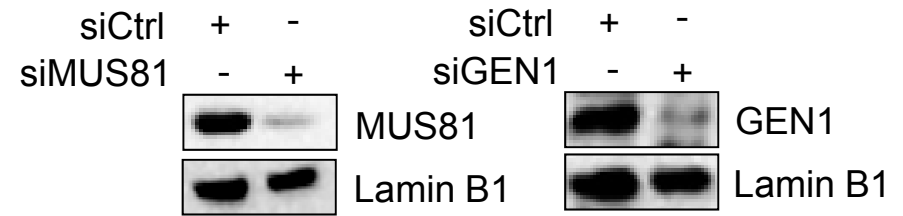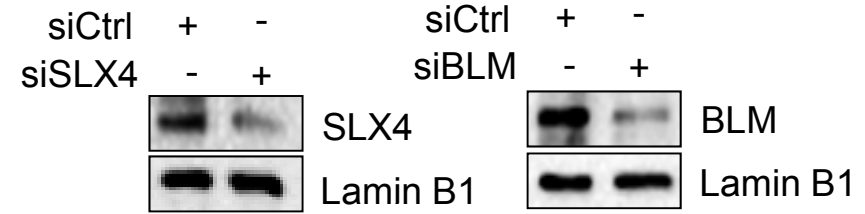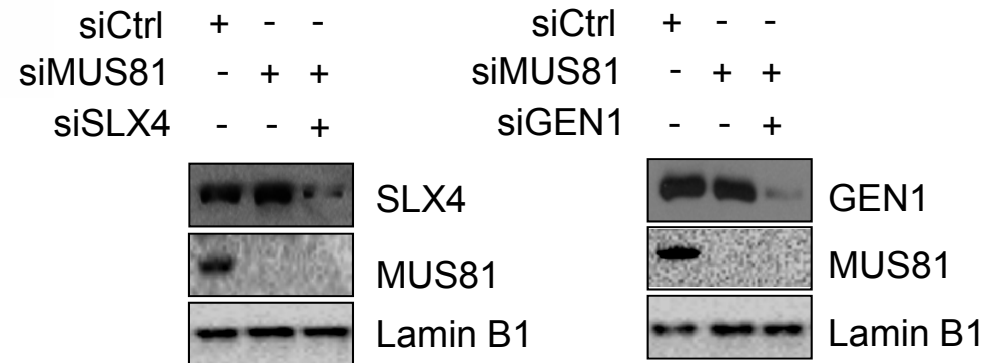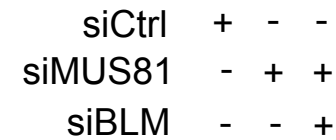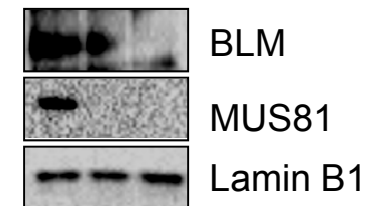

Figure S9

Supplement: Figure S9 — Viability of cells depleted of different SSEs or BLM after replication stress induced by CHK1 inhibition. (A) GM01604 cells were transfected with siRNAs directed against GFP (siCtrl) or against the indicated SSEs or BLM. (B) Evaluation of cell death in cells were transfected with a combination of the indicated RNAi oligos. In all cases, cells were treated 48 h post-transfection with 400 nM UCN-01 alone or in combination with 2 mM HU for 6 h, followed by recovery for 18 h in drug-free medium prior to evaluation of cell death by the LIVE/DEAD assay. Graph shows data presented as means +/− SE from three independent experiments. Western blot panels show actual depletion levels obtained for each of the depletion analyzed in (A) and (B). (PDF) [file pgen.1003910.s009.pdf]
