# Supplementary material for: Survival of the Replication Checkpoint Deficient Cells Requires MUS81-RAD52 Function
Source: PLoS Genet. 2013 Oct 31;9(10):e1003910. doi: 10.1371/journal.pgen.1003910 (PMC3814295; doi:10.1371/journal.pgen.1003910)
Supplement: Figure S10 — Viability of cells depleted of RAD52 in combination with different SSEs or BLM (A) GM01604 cells were transfected with siRNAs directed against RAD52 and MUS81, alone or in combination with RNAi oligos against the SSEs SLX4 and GEN1 (B) Evaluation of cell death in cells were transfected with a combination of the indicated RNAi oligos. In all cases, cells were treated 48 h post-transfection with 400 nM UCN-01 alone or in combination with 2 mM HU for 6 h, followed by recovery for 18 h in drug-free medium prior to evaluation of cell death by the LIVE/DEAD assay. Graph shows data presented as means +/− SE from three independent experiments. Western blot panels show actual depletion levels obtained for each of the depletion analyzed (B); For what concerns efficiency of the oligos used for multiple depletion analyzed in panel (A), refer to Figure 3D. (PDF) [file pgen.1003910.s010.pdf]

A

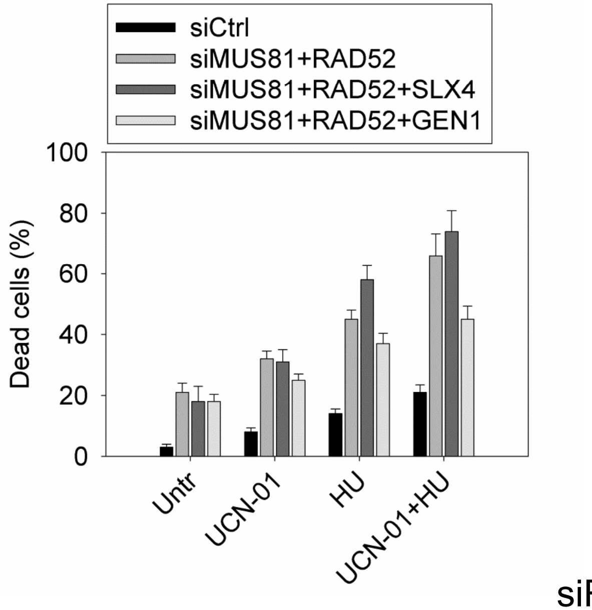

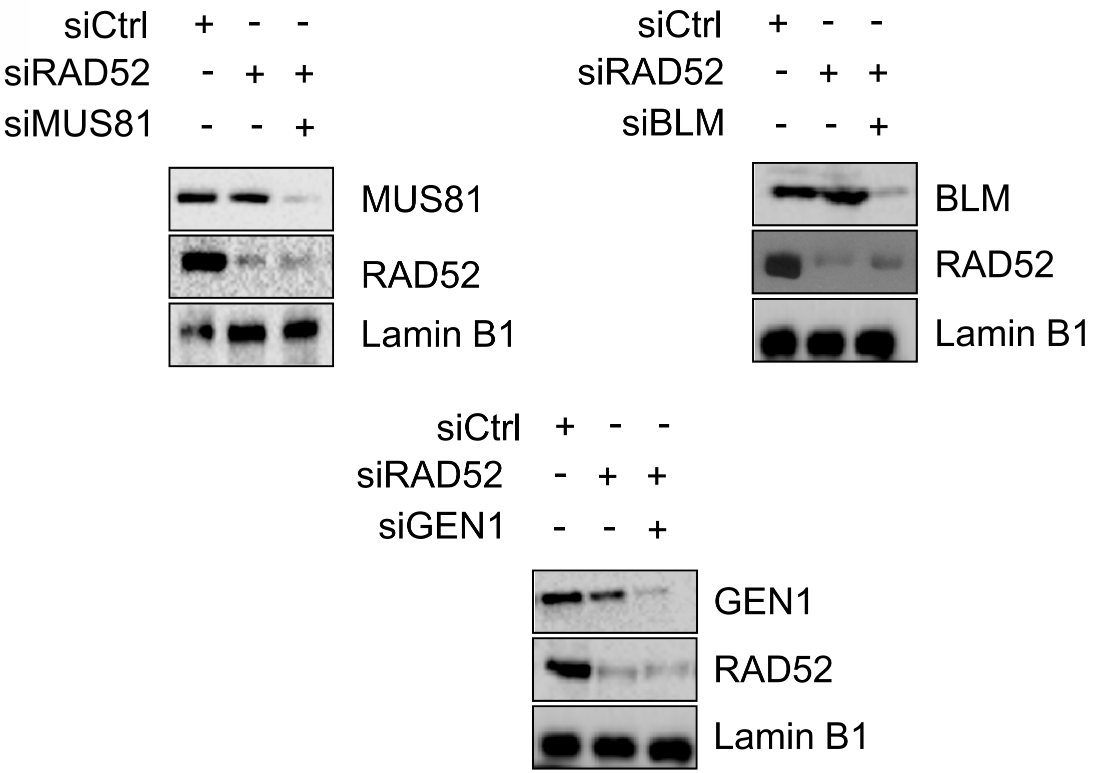

B

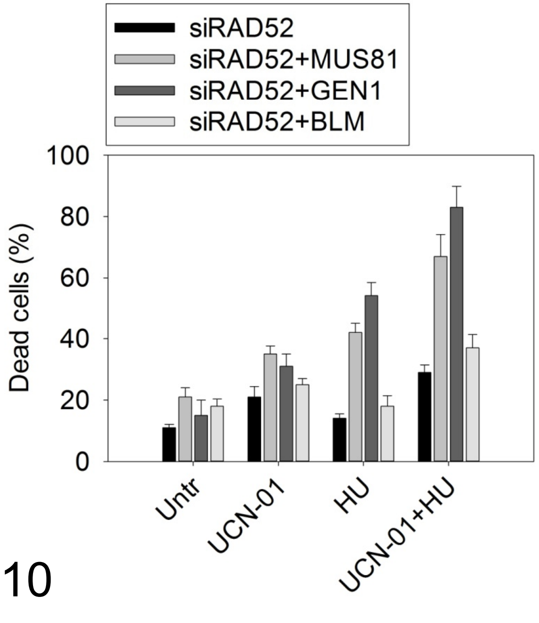

Figure S10
