# Supplementary material for: Survival of the Replication Checkpoint Deficient Cells Requires MUS81-RAD52 Function
Source: PLoS Genet. 2013 Oct 31;9(10):e1003910. doi: 10.1371/journal.pgen.1003910 (PMC3814295; doi:10.1371/journal.pgen.1003910)
Supplement: Text S1 — The file contains supplementary methods for immunofluorescence; evaluation of S-phase content, flow-cytometry analysis, and site-directed mutagenesis. (DOC) [file pgen.1003910.s011.doc]

**Supplementary Materials and Methods**

**Immunofluorescence**

Immunofluorescence microscopy was performed on cells grown on coverslips as described (Franchitto et al., 2008). For immunostaining of RAD51 cells were incubated at RT for 2h with a rabbit polyclonal anti-RAD51 (Calbiochem) in 1% BSA/PBS. Specie-specific Texas Red-conjugated secondary antibodies (Jackson Immunoresearch) were applied for 1h at RT, followed by counterstaining with 0.5μg/ml DAPI in DABCO. For each time point, at least 200 nuclei were examined by two independent investigators and foci were scored at 60×. Only nuclei showing more than five bright foci were counted as positive. Parallel samples incubated with either the appropriate normal serum or only with the secondary antibody confirmed that the observed fluorescence pattern was not attributable to artifacts. Coverslips were analysed through a microscope (Leica) equipped with a charge-coupled device camera (Photometrics). Images were acquired as greyscale files using the Methaview software (MDS Analytical Technologies) and then processed using Photoshop (Adobe).

**Evaluation of S phase cells**

To quantify S phase cells, GM01604 were pulse labeled for 30min with 30mg/ml BrdU and then samples were processed for immunodetection of BrdU incorporation essentially as described previously (Pichierri et al., 2001). For each time point, at least 500 interphase cells were scored to evaluate the percentage of labeled nuclei. Only nuclei displaying more or less uniform BrdU labeling in the entire volume were considered to be actively replicating. The percentage of cells undergoing DNA synthesis at each time point was calculated as a fraction of the treated cells versus untreated controls.

**Cell cycle analysis by flow cytometry**

GM01604 cells were transfected with siRNAs directed against GFP (control) or against MUS81 (siMUS81), then synchronized in G1/S by starvation in DMEM 0,1% FBS for 48h followed by 24h of release in DMEM 15% FBS. Then cells were treated with 400nM UCN-01 and/or for 6h with 2mM HU prior to be recovered for indicated time. Samples were collected and processed for flow cytometry as described (Franchitto et al., 2008) and data analysed with CellQuest software.

**Site-directed mutagenesis**

The T309A and T309D mutations were generated using the Stratagene XLII mutagenesis kit, according to the manufacturer’s directions. Briefly, mutations were introduced with specific mutagenic primer pairs and using the a plasmid for expression of the T7-tagged wild-type RAD51 ORF in human cells as template. The presence of the desired mutations and absence of unwanted DNA changes was confirmed by sequencing.
